# An Evidence-Based Systematic Review of Human Knee Post-Traumatic Osteoarthritis (PTOA): Timeline of Clinical Presentation and Disease Markers, Comparison of Knee Joint PTOA Models and Early Disease Implications

**DOI:** 10.3390/ijms22041996

**Published:** 2021-02-17

**Authors:** Christine M. Khella, Rojiar Asgarian, Judith M. Horvath, Bernd Rolauffs, Melanie L. Hart

**Affiliations:** G.E.R.N. Center for Tissue Replacement, Regeneration & Neogenesis, Department of Orthopedics and Trauma Surgery, Faculty of Medicine, Medical Center—Albert-Ludwigs-University of Freiburg, 79085 Freiburg im Breisgau, Germany; christine.mounir86@gmail.com (C.M.K.); r0jiar.asgarian@gmail.com (R.A.); judith.m.horvath@gmail.com (J.M.H.); bernd.rolauffs@uniklinik-freiburg.de (B.R.)

**Keywords:** chondrocyte, articular cartilage, osteoarthritis, post-traumatic osteoarthritis, immunomodulation, acute, subacute, chronic, inflammation, early PTOA, cartilage repair, clinical, knee trauma, knee joint, injury, inflammatory cytokines, synovial fluid, in vitro models, cartilage, IL-1β, TNF-α, IL-6, IL-17, complement, Bradford Hill, early disease

## Abstract

Understanding the causality of the post-traumatic osteoarthritis (PTOA) disease process of the knee joint is important for diagnosing early disease and developing new and effective preventions or treatments. The aim of this review was to provide detailed clinical data on inflammatory and other biomarkers obtained from patients after acute knee trauma in order to (i) present a timeline of events that occur in the acute, subacute, and chronic post-traumatic phases and in PTOA, and (ii) to identify key factors present in the synovial fluid, serum/plasma and urine, leading to PTOA of the knee in 23–50% of individuals who had acute knee trauma. In this context, we additionally discuss methods of simulating knee trauma and inflammation in in vivo, ex vivo articular cartilage explant and in vitro chondrocyte models, and answer whether these models are representative of the clinical inflammatory stages following knee trauma. Moreover, we compare the pro-inflammatory cytokine concentrations used in such models and demonstrate that, compared to concentrations in the synovial fluid after knee trauma, they are exceedingly high. We then used the Bradford Hill Framework to present evidence that TNF-α and IL-6 cytokines are causal factors, while IL-1β and IL-17 are credible factors in inducing knee PTOA disease progresssion. Lastly, we discuss beneficial infrastructure for future studies to dissect the role of local vs. systemic inflammation in PTOA progression with an emphasis on early disease.

## 1. Introduction

Osteoarthritis (OA) is the most prevalent form of joint disease and one of the leading causes of disability affecting 630 million people worldwide [1]. While OA is considered an age-related disease, it also affects younger individuals with 8% of the population aged 18–44, 30% of the population aged 45–64 and 50% of individuals over 65 years of age having OA. Joint trauma is a major cause of this degenerative disease and, astoundingly, 23% to 50% of the people that suffer a trauma to the knee joint eventually develop post-traumatic osteoarthritis (PTOA) [2,3,4,5,6,7,8]. Those with prior knee joint trauma are 3 to 6 times more likely to develop PTOA and were diagnosed 10 years earlier than those without any history of an injury [1,9,10].

Inflammation has been shown to be play a role in the pathogenesis of OA [11,12,13] or PTOA [14,15,16,17,18] in general. While other reviews suggest a general timeline of the pathogenic response following joint injury, they do not (a) focus solely on a particular joint (i.e., acute knee joint injury), and/or (b) do not provide a detailed clinical inflammatory timeline and sequence of events of inflammatory and other mediators present in the synovial fluid (SF), serum, plasma and urine following knee trauma. Moreover, a detailed review that relates the available detailed knowledge derived from basic science studies, which simulate injury and induce inflammation, with data of clinically orientated studies is lacking but would be beneficial to identify types and concentrations of inflammatory mediators in post-traumatic patient tissues related to the knee joint and/or fluids. The objective of this review was, therefore, to develop a timeline of events after knee trauma from early acute inflammation to development of final end-stage clinical PTOA and to compare the clinical stages of inflammation to the in vivo and in vitro/ex vivo models of injury and/or inflammation. The aim was to identify key inflammatory inducers of knee PTOA and to determine if the models as well the concentrations of inflammatory cytokines commonly used to induce inflammation are representative of clinical knee PTOA. We also discuss beneficial infrastructure for future studies to dissect the role of local vs. systemic inflammation in PTOA progression of the knee joint with an emphasis on early disease.

## 2. Methodology

In preparing this systematic review, eligible articles were identified using PubMed, Medline, Cochrane Library, Google Scholar, Web of Science databases, and by hand-searching. The following inclusion criteria were used: (1) peer-reviewed studies that were published in the English language and, unless otherwise stated, (2) clinical samples that were obtained from the knee joint or from the SF, serum, plasma or urine following acute knee trauma from patients having no history of a previous knee injury; (3) in vivo PTOA models of the knee joint that applied insult/injury and/or inflammatory cytokines (chemically induced models of injury were excluded), and (4) in vitro chondrocyte and cartilage ex vivo explant models that used cells, cartilage, SF and/or the joint capsule obtained or isolated exclusively from the knee joint.

Each clinical study was assessed for quality using the National Institute of Health (NIH) Study Quality Assessment Tool, which can be accessed at URL: www.nhlbi.nih.gov/health-topics/study-quality-assessment-tools (accessed on 14 November 2020). This tool is widely used for critically appraising cohort and cross-sectional studies and consists of 14 questions, of which most can be answered with “yes” (assigned a value of 1) or “no” (assigned a value of 0). Other possible answers were not reported (NR) and not applicable (NA). Based on the total score, the quality assessment was defined as 0–5 (poor), 6–9 (fair) or 10–14 (good).

The Bradford Hill Framework provides nine criteria for establishing epidemiologic evidence of a causal relationship between an exposure and an observed effect [19]. We used this framework to evaluate the relationship between the presence of inflammatory markers (IL-1β, TNF-α, IL-6 or IL-17) after knee trauma and PTOA disease progression. The criteria, which are widely used in public health research and have stayed virtually unchanged since it was first published, are as follows: strength of the association, consistency of findings, specificity of the association, temporal sequence of association, biological gradient, biological plausibility, coherence, experimental evidence and analogy. Using this framework, we determined whether IL-1β, TNF-α, IL-6, and/or IL-17 led to a convincing, credible or probable causal PTOA disease effect, or whether the evidence was suggestive, limited, or inconclusive. The evidence collected for each viewpoint is presented with a final judgement as to whether the nine criteria were fulfilled or not.

Because we noted that each clinical assessment chose a different time range for the collection of clinical samples, in this review we defined acute inflammation as inflammation occurring directly after knee injury and lasting for up to 2 weeks, subacute inflammation as the time between acute and chronic inflammation lasting 2 to 6 weeks, and chronic inflammation, as prolonged inflammation exhibited by a significant increase in inflammatory markers, lasting for prolonged periods from greater than 6 weeks to years following knee injury [20].

## 3. Results of the Clinical Studies That Measured Inflammatory and Other Key Biochemical Biomarkers in Patients Who Have Had Knee Trauma

The quality assessment results of the clinical knee injury studies included in this systematic review demonstrate that all of the included studies were of good or fair quality (Table 1). The main limitations and reasons for scores of zero were due to a lack of a power calculation or justification of sample size, the absence of a blinded outcome assessment, and/or not considering possible confounding factors in the analysis. The confounding factors are listed in Table 2 along with the types of injury, the group in which injury samples were compared, the post-traumatic phase (acute, subacute, chronic, and/or PTOA), the markers measured after knee trauma, the age, cohort size, and the number of males/females that were included in the studies.

Samples were measured in the synovial fluid, serum/plasma and/or urine within 24 h and up to many years following knee trauma and ranged from the acute, subacute, and chronic phases of inflammation after knee trauma to clinical PTOA of the knee joint. Most studies were cross-sectional studies in which only one sample was taken from each patient. The type of reported knee trauma was mostly caused by anterior cruciate ligament (ACL) and medial collateral ligament injuries [23,24,25,26,27,28,29,30,31,32,34,35,36,37,38] or intra-articular fracture [21,22,23,28,33,36]. Fewer injuries were due to patellar dislocation, rotational knee trauma, knee contusion, medial meniscus injury, or posterior cruciate ligament injury [23,27,28,36,39]. Some studies included controls and others did not (Table 2). A few studies also investigated the contralateral uninjured knee joints or included OA, post-meniscectomy, hydrarthrosis, or chronic arthritis samples for comparisons. Most of the studies consisted of a larger percentage of males (range 59–100%) [21,22,23,24,25,26,27,28,31,32,34,35,36,37,38] rather than female subjects, with only a few studies having a majority of females in their cohort that reached up to 55% [33,39]. The age of patients having a knee injury varied from 13 to 70 years old [21,22,23,24,25,26,28,29,30,31,32,33,34,35,36,37,38,39] and had a mean age of 29 years [21,22,23,24,25,26,28,30,31,34,35,36,37,38].

**Figure 1:**
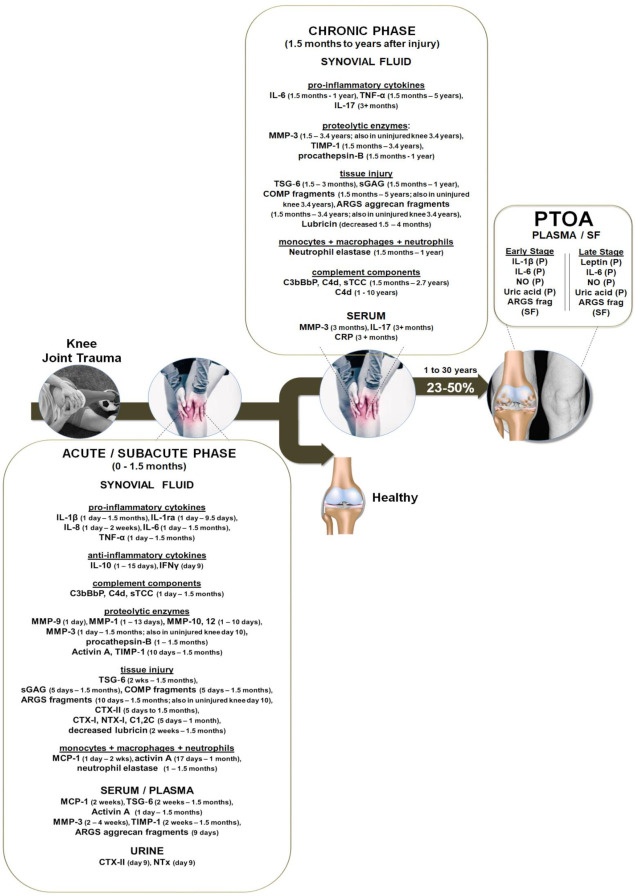
PTOA development after knee joint injury. Following knee joint injury, an immediate acute phase of inflammation occurs which continues for up to 2 weeks and is typically followed by a subacute phase, lasting up to 1.5 months, exhibited by a primary high wave of inflammatory cytokines that decrease over time but remain higher than uninjured controls. Likewise, other mediators, including complement components, neutrophil and macrophage-associated factors, MMPs, degradative proteolytic enzymes and ECM fragments increase in the synovial fluid, while lubricin decreases, all indicative of early, and possibly irreversible, damage to the cartilage tissue. When inflammation is not resolved, cartilage degradation continues to be associated with a low-grade chronic phase of inflammation characterized by decreased, but higher than normal, levels of pro-inflammatory cytokines IL-6, TNF-α and IL-17. Although these inflammatory mediators are lower than what may be observed in OA or RA patients, this sustained low-grade inflammation is still clearly present, significantly higher than healthy controls, and correlates with degenerative effects, all of which promote joint pathology. A long-term inflammatory status persists, leading to a systemic effect (presence of biomarkers in the blood and/or uninjured knee) accompanied with alterations in joint function leading to PTOA. This figure was composed based on all of the clinical data discussed in this review that measured the concentrations of these markers at the (mean) time or range of times (using the data of multiple studies) post-injury showing, with the exception of lubricin which was decreased, significantly high levels of these markers at the indicated time points.

Although differences in the timescale and the choice of markers may make comparisons across clinical studies difficult, we simplified the resulting data by (a) including such information in the text and (b) by dividing the clinical studies into different sections to create a timeline of events that occur after knee trauma (Figure 1). We first present studies representative of localized knee joint inflammation, followed by studies that measured proteolytic enzymes and tissue injury markers. We then present studies that demonstrate the systemic effects of injury, and finally discuss the inflammatory factors that were measured in clinically diagnosed cases of PTOA due to knee joint trauma.

### 3.1. Continuous Localized Inflammation from Months to Years after Knee Trauma

As pro-inflammatory and anti-inflammatory cytokines are the major immunoregulatory molecules that control the immune response and could dictate the direction towards or against PTOA progression, we first focused on studies that measured these cytokines after knee trauma. Haller et al. showed that, compared to uninjured knees, the SF collected 24 h after acute tibial plateau fracture contained significantly higher levels of IL-1β, IL-6 and IL-8, as well as monocyte chemoattractant protein-1 (MCP-1), also known as CCL2, which regulates the migration and infiltration of monocytes and macrophages [21]. While anti-inflammatory cytokines IL-4 and IL-13 were not detectable at any time point, anti-inflammatory and immunoregulators IL-10 and IL-1 receptor antagonist (IL-1ra), the natural inhibitor or regulator of IL-1β, were locally and significantly increased 24 h after knee injury. Approximately half of those patients that were reassessed at a mean of 9.5 days after injury, which showed that the concentrations of IL-6, IL-8, MCP-1, IL-10 and IL-1ra concentrations continued to remain elevated in the injured vs. uninjured knees. Interestingly, they did not detect any differences in SF cytokine concentrations between low vs. high energy injuries 24 h after fracture, suggesting a similar immediate immune response in tibial plateau fractures despite the level of impact energy. Similar to the aforementioned study, Watt et al. also showed that MCP-1 was significantly elevated in both the SF and serum in samples taken within 8 weeks (median 17 days) of knee injury vs. controls [28] suggesting acute phase infiltration of monocytes and macrophages into the knee joint. This group also showed that, IL-6, Activin A (a member of the TGF-β superfamily that is closely related to TGF-β1 and controlled by fibroblast growth factor 2 and NF-κB [40]), and TSG-6 (a product of TNF-α gene 6) that binds to complement proteins, hyaluronic acid and CXC and CC chemokines [41]) were all significantly elevated in the SF vs. controls and, while the concentrations decreased over time, the majority of patients still had IL-6, Activin A, and TSG-6 present 1.5 months after injury with levels higher than control values [28]. Activin A and TSG-6 have both been strongly associated with rapid knee OA progression [42,43]. In this study, IL-1β was below the limit of detection for all but one individual. Higher levels of SF IL-6 associated with worse clinical symptoms and loss of function, based on the Knee Injury and Osteoarthritis Outcome Score 4 (KOOS_4_). Even though the levels of IL-6 increased with the severity of injury, the inflammatory response in all types of knee injuries was similar, suggesting that the inflammatory response is comparable across different types of injuries. Higuchi et al. similarly showed that IL-6 was significantly elevated compared to controls at a mean of 6 months (range of 2–140 weeks) after injury [31]. Moreover, SF matrix metalloproteinase-3 (MMP-3) concentration significantly correlated with the IL-6 concentration among the various cytokines, but did not correlate with IL-1β or TNF-α.

Similar to the aforementioned study [21], Swärd et al. showed that injured patients with hemarthrosis, which is bleeding into the joint cavity, exhibited an increase in IL-1β, TNF-α, IL-6 and IL-8 in the SF 24 h after injury, followed by a time-dependent decrease thereafter, with IL-8 remaining significantly higher than age- and gender-matched healthy controls up to 15 days and IL-1β, TNF-α and IL-6 up to 23 days after injury [23]. Catterall et al. demonstrated the presence of IL-1β at both time points measured (i.e., 15 to 47 days post-injury) [27]. Bigoni et al. screened SF collected after various time points, including within 48 h after injury, between 3 and 15 days, 15 days and 3 months, or more than 3 months following knee injury [24]. Within 48 h of injury, the SF concentrations of IL-1β, IL-6, and IL-8 were significantly increased vs. healthy controls. IL-1β steadily decreased after 48 h and between days 3 to 15 days post-injury, and from that point on it was comparable to control values. IL-8 also gradually decreased and was comparable to controls in the 15 day to 3 months post-injury group. IL-6 remained high up to 15 days post injury and, although it decreased, it continued to be present in patients with chronic symptoms (defined as 3 months or more post injury) and was significantly higher than controls. TNF-α progressively and significantly increased with time after injury and was still elevated up to 3 or more months post injury. While IL-10 was not compared to controls in this study, at all time points it was comparable to healthy controls reported in another study [25]. Interestingly, in the 0–48 h post-injury group, IL-1ra was significantly lower than the control group and did not increase at any of the time points. While the study of Lattermann et al. did not have a control group, they also showed that IL-1ra significantly decreased from day 5 (range 2–8 days) to day 13 (range 9–20 days) post injury and continued to decrease 28 days (range 15–45 days) post injury [29].

Irie et al. measured the concentration of SF cytokines at several different time points (within 24 h, 2–3 days, 4–6 days, 7–9 days, 10–14 days, 15–21 days) after injury, and determined whether cytokine concentrations were comparable or higher than patients already with chronic arthritis in the knee [25]. In line with the aforementioned studies, this study demonstrated a comparable immediate inflammatory response in which the levels of cytokines TNF-α, IL-1β, IL-6, IL-8, IL-1ra, and IL-10 were significantly elevated within 24 h and then steadily decreased thereafter. Notably, despite a decrease in cytokines over time, concentrations were higher than in chronic arthritis patients with IL-1ra elevated up to 6 days and IL-10 up to 9 days post-trauma. With the exception of the 10–14-day injury group, IL-1β was significantly higher than in chronic arthritis patients at all of the other days and remained significantly higher up to 21 days post-injury. TNF-α was also significantly higher than in chronic arthritis patients and at all time points up to 3 weeks post-injury. While IL-6 concentration values were exceedingly high compared to the other cytokines measured in this study, the concentrations of IL-6 only exceeded those of chronic arthritis patients during the first 3 days following knee trauma.

Other studies have also shown that local inflammation persists and develops into chronic inflammation [26,30,31,32,33,34,35,36]. Thus, IL-17 concentrations were significantly higher in the SF for 3 or more months after injury compared to healthy controls [26]. Elsaid et al. showed that, when SF aspirations were taken at a mean of 3 months (range of 32–364 days) after injury, patients had elevated levels of IL-1β, IL-6 and TNF-α, compared to uninjured knees [26]. The concentrations of individual cytokines were also assessed in the injured knee and plotted against the time of collection after injury, which showed that IL-1β was present 1 to 3 months post-injury in 50% of the patients andTNF-α in 60% of the patients 1 to 6 months after injury. Interestingly, IL-6 and the catabolic enzymes procathepsin-B and neutrophil elastase were detectable from 1 month up to 1 year post injury in the injured knees in 95%, 100% and 80% of patients, respectively.There were no detectable levels of these markers in SF samples taken from the contralateral joints. Struglics et al. showed that the concentrations of cytokines IL-6, IL-8, IL-10, IFN-γ, TNF-α and and Alanine–Arginine–Glycine–Serine (ARGS) aggrecan fragments fragment in SF collected from the injured patients at the initial 0–6 weeks (mean of 9 days) time point were significantly higher than those of healthy subjects but their levels steadily decreased over time, with SF TNF-α remaining elevated for up to 5 years after injury [32]. Moreover, over the 5-year period, ARGS aggrecan fragments concentrations in SF correlated with all of the SF cytokines measured. Using a mixed variance components model with values adjusted for differences in age, sex, and body mass index (BMI), this group estimated that TNF-α has a half-life of 3.6 years in the SF, while IL-6 has a half-life of 0.9 years in the SF and, astonishingly, a half-life of 45 years in the serum.

Hence, all of the studies discussed here point to continued subacute and chronic inflammation in the knee joint after knee trauma with multiple studies confirming that TNF-α and IL-6 remain significantly elevated months to years after knee trauma and at comparable concentrations to chronic arthritis patients.

### 3.2. Activation of the Complement System Correlates with Inflammation Months to Years after Knee Trauma

Since it is believed that the crucial and driving event in the development of PTOA is a sustained early inflammatory reaction after trauma or acute injury [14,15,16,18] (this review), it is also important to explore a possible contribution of the complement system to knee PTOA. The 3 distinct but overlapping complement pathways, referred to as the classical, alternative and lectin complement pathways, are activated through various mechanisms that result in a proteolytic cascade of events that generates potent pro-inflammatory molecules and leukocyte chemoattractants, which (i) recruits inflammatory cells such as neutrophils, monocytes, macrophages and T lymphocytes, and (ii) leads to the production and assembly of the terminal membrane attack complex (MAC), sometimes referred to as the terminal complement complex (TCC), a cytolytic protein complex that activates destroys cells. While this complex network of activating, and its regulating proteins, is needed for clearance of pathogens and dead or dying cells, and thereby helps support repair of injured tissues, it can also lead to detrimental effects on the host by exerting pathological mechanisms that promote tissue damage and chronic inflammation [36,44,45,46]. 

One study has highlighted complement’s role in chronic inflammation and the possible progression of PTOA in the knee [36]. This group measured the SF levels of C4d, C3bBbP, and soluble TCC (sTCC) from those with a recent knee injury (defined as 1–83 days after injury) or an old injury (defined as 1–37 years after injury) with 8% of those patients diagnosed with PTOA, as well as from healthy controls from another cohort and OA, RA and pyrophosphate arthritis (PPA) patients. Patients with a recent knee injury or OA displayed similar increased levels of C4d, C3bBbP and sTCC in the SF which were significantly higher than those of healthy controls. Moreover, 98% of patients with recent knee injuries had hemarthrosis, suggesting that complement components present in the SF after initial knee trauma may stem from intra-articular bleeding. The concentrations of pro-inflammatory cytokines TNF-α, IL-6 and IL-1β positively correlated with the levels of complement factors in patients with a recent injury, with the strongest correlation between SF TNF-α and C4d levels. While complement components decreased over time, C4d remained elevated in SF that was aspirated up to 10 years after knee injury, demonstrating the continuous chronic activation of the complement system following trauma to the knee.

In addition to a potential role in PTOA development, several groups have also shown that the complement cascade is involved in the pathogenesis of both early and late-stage OA in the knee [36,47,48,49,50,51,52]. C3a and sTCC were significantly higher in the SF from patients with early-stage knee OA (having symptoms less than 1 year) compared to healthy individuals [49,51] and complement transcripts and/or activation components C3/C3a, C4a, C4d, C3bBbP, factor B, and sTCC have been detected at high levels in the SF, synovial membrane and in the cartilage of patients with OA in the knee joint [36,47,48,49,53]. Recent evidence suggests that all three complement pathways are capable of being activated within the knee joint in both early and late knee OA and that the synovial membrane is the main source of complement activation rather than the articular cartilage tissue [50]. Patients with OA also exhibited high concentrations of lectin complement pathway components, including mannose bindin lectin (MBL), H-ficolin, M-ficolin, mannan-binding lectin serine protease- 2 (MASP-2), and MASP-3, which were higher in the plasma than the SF [54], and while the levels were significantly lower than patients with RA, this data suggests a possible role of the lectin pathway in promoting chronic disease in knee OA. Moreover, inhibitors of the complement system, including factor H, C4-binding protein, C1 inhibitor and clusterin, were significantly decreased in both early- and end-stage OA vs. healthy synovial membranes [51], suggesting a lack of complement system regulation. Thus, complement could be a major contributor to not only injury-related acute inflammation but also chronic inflammation and possibly PTOA pathogenesis.

### 3.3. Release of MMPs, ECM Components and Damage to the Cartilage Collagen Network Months to Years after Knee Trauma

In addition to increased levels of inflammatory cytokines after knee joint injury, the expression of MMPs, the release of ECM components, and the degradation of type II collagen in articular cartilage tissue is increased compared to the controls. In the same set of patients from [21], Haller et al. showed that MMP-1, 3, 9, 10, 12 were initially elevated in the acute samples (within 24 h after injury) with MMP-1, 3, 10, 12 remaining elevated at the second aspiration a mean of 9.5 days (range of 3–21 days) after injury [22]. Lattermann et al. also showed that MMP-1 significantly increased from day 1 to day 13 after injury [29]. Watt et al. demonstrated that, compared to controls, MMP-3 and TIMP-1 were significantly elevated in the SF samples that were obtained within 8 weeks (median 17 days) of knee injury and, while the concentrations decreased over time, the majority of patients still had higher than control concentrations of MMP-3 and TIMP-1 1.5 months after injury [28]. Higher levels of SF MMP-3 and TIMP-1 also associated with the presence of hemarthrosis (moderate or severely blood-stained SF) and the severity of injury. In line with the aforementioned studies, Higuchi et al. similarly showed that MMP-3 and TIMP-1 remained highly elevated in the SF at a mean of 6 months (range of 2–140 weeks) after injury compared to healthy controls [31].

Lubricin, a glycoprotein secreted by synoviocytes as well as superficial zone chondrocytes found in the SF that covers the superficial layer of articular cartilage and provides boundary lubrication in articular joints, is also affected by knee injury. Catterall et al. showed that SF lubricin levels were significantly decreased in injured knees when comparing levels at baseline (mean 15.2 ± 7.2 days) to the follow-up (mean 47.6 ± 12.4 days) [27]. Similarly, Elsaid et al. showed that the levels of lubricin significantly decreased in the injured knee 2–4 months, compared to the contralateral uninjured knee joint [26]. While lubricin returned to normal levels within 1 year, there was a significant inverse correlation between high SF TNF-α and low SF lubricin concentrations and between low SF lubricin and the high release of sulfated glycosaminoglycan (sGAG), which also remained significantly elevated from 1 month to 1 year after injury in injured vs. uninjured knee joints.

In addition to synovial inflammatory biomarkers and MMPs, major ECM cartilage degradative products increased rapidly upon injury, and while they decreased in the subacute to chronic phases, their levels remain significantly higher than healthy controls. Whereas the following two studies did not include controls, Lattermann et al. showed that sGAG and the non-collagenous ECM cartilage oligomeric matrix protein (COMP) fragments were highest in the SF during the first two weeks of injury but started to decrease at a mean of 28 days post-injury, while C-telopeptide fragments of collagen type II (CTX-II) increased over time from the acute (day 5 and day 14) to the subacute (day 28) phase [29]. Catterall et al. similarly showed that the concentrations of sGAG and ARGS aggrecan fragments decreased from 15.2 ± 7.2 days to 47.6 ± 12.4 days after injury, while CTX-II, as well other collagen I and II collagen fragments, including C-terminal crosslinked telopeptide type I collagen (CTX-I), N-terminal telopeptides of type I collagen (NTX-I) and C1,2C significantly increased [27]. In line with these studies, Lohmander et al. showed that CTX-II peaked hours after injury and, while concentrations gradually decreased over time, CTX-II remained present and significantly higher in the SF vs. healthy uninjured controls 1 week, 2.5 months, 1 year and even up to 2.7 years after injury [34]. Similarly, Struglics et al. demonstrated that SF COMP fragments were significantly higher than controls at all time points investigated (i.e., 9.1 days, 3.6 and 7 months, and 1, 2, and 5 years after injury), which positively correlated with SF IL-6, IL-8, TNF-α, IFNγ and IL-10, with SF and serum ARGS aggrecan fragments and urine CTX-II and NTX-I levels [37].

In another study, Struglics et al. showed that the ARGS neoepitope was increased in the SF 4–7 months after injury compared to the controls [32], whereas Lohmander et al. demonstrated that the concentration of the ARGS aggrecan fragments in the SF peaked hours after injury, significantly increased from 2 weeks to 10 weeks after injury, and then decreased gradually during the first year after injury, but then leveled off with concentrations remaining significantly elevated vs. healthy controls 1 week, 2.5 months, 1 year and up to 2.7 years after injury [35]. Dahlberg et al. demonstrated that the SF concentrations of ARGS and COMP fragments as well as MMP-3 and TIMP-1 were greater than healthy controls at 10 days post-injury and, while the concentrations of markers in the injured knee decreased with increased time after injury, the concentrations of COMP fragments, MMP-3 and TIMP-1 were still higher than healthy controls 3.4 years after injury [30].

These studies indicate that the injured knee joint continues to be exposed to the degradative action of catabolic enzymes during the early acute, to subacute and chronic phases following injury, and that this exposure correlates with measurable inflammation. While the above studies demonstrate a temporal decrease in tissue injury markers, they point to a sustained post-traumatic inflammation, which is associated with a long-term, continuing degradation of the cartilage matrix after knee joint injury, suggesting a lasting role in progression to PTOA.

### 3.4. Systemic Measurable Effects after Knee Trauma

While the inflammatory response following joint injury may in part be localized to the knee joint, studies investigating inflammatory components in the blood (serum/plasma), contralateral uninjured knee and urine have also demonstrated the presence of systemic subacute and chronic inflammation following knee trauma. Sarafan et al. showed that IL-17 was present in the serum (and SF as already discussed) three or more months after knee trauma. OA patients had significantly higher serum IL-17 concentrations than patients with intra-articular knee joint fractures, with the healthy control group exhibiting the lowest IL-17 values [33]. Other studies have similarly shown both circulating and SF IL-17 in patients diagnosed with early- or late-stage knee OA compared to healthy controls and that synovia IL-17 levels correlate with knee OA severity [35,55,56,57,58,59,60].

Multiple studies showed that CRP concentrations in the serum significantly exceeded the levels in SF, beginning 2 to 3 months following knee injury [27,28,33]. CRP levels were equally present in the serum and SF of patients having either OA or an intra-articular fracture with associated pain for at least three months and, in both cases, CRP levels were significantly higher than in the controls [33]. Catterall et al. showed that, while CRP levels decreased over time in both the serum and SF from 15.2 ± 7.2 days to 47.6 ± 12.4 days after injury, there was a significant correlation between SF and serum CRP [27]. Watt et al. demonstrated a similar correlation between serum and SF CRP up to 3 months compared to the baseline samples obtained within 2 months after injury [28].

Struglics et al. showed that the urine concentrations of the collagen fragments CTX-II and NTX-I were initially increased at the baseline point (mean 9 days after injury; range 0–6 weeks) in the patients having knee injury, but levels decreased with time after injury [32]. Catterall et al. found significant correlations between the serum and SF concentrations of CTX-I and NTX-I and MMP-3 1.5 months after injury [27]. Watt et al. showed that at the baseline visit (taken within 8 weeks of knee injury; median 17 days), like the concentrations found in the SF, serum/plasma concentrations of MMP-3, TIMP-1, TSG-6, MCP-1, and Activin A were also significantly elevated compared to controls [28]. In fact, TSG-6 levels remained constant in the serum, while MMP-3 continued to increase in the serum up to 3 months after baseline taken within 2 months of injury, suggesting that MMP-3 was still systemically present up to 5 months post-injury. Dahlberg et al.’s data demonstrated that the contralateral uninjured also had significantly higher concentrations of MMP-3 as well as ARGS and COMP fragments compared to healthy controls at 10 days and 3.4 years after injury [30]. Moreover, the SF concentrations of MMP-3 and COMP fragments were similar in the injured and the contralateral uninjured knee 3 months and 3.4 years after knee injury, further emphasizing systemic long-term effects of knee injury. Struglics et al. also showed a correlation between the concentrations of the ARGS neoepitope in the SF and serum [32]. These studies highlight the systemic long-term effects of knee trauma.

Pengas et al. showed that, even up to forty years after open total meniscectomy (an outdated surgical method of repairing meniscal tears), SF MMP-3 levels remained high and, while sGAG levels were low, both correlated with the radiographic OA score. Moreover, serum MMP-3 correlated with MMP-3 SF levels in the operated knee. Interestingly, they showed that the quality of life could be predicted 40 years after surgery using the pre- and post-surgery concentrations of MMP-3 and sGAG and patient age [61]. Together, these studies [22,27,28,29,30,31,61] indicate the importance of MMP-3 as a reflective systemic biomarker of degradative activity after knee trauma that is measurable in both injured and uninjured knee joints and even in serum.

Importantly, these data suggest that, whereas the inflammation caused by injury originated from a single knee injury, the inflammatory effects lead to a systematic response, suggesting that certain serum markers could potentially be assessed along with other clinical assessments of joint inflammation and injury in the subacute to chronic phases to post-traumatically monitor systemic knee injury and possible progression to PTOA.

### 3.5. Local and Circulating Inflammatory Cytokines and Other Biochemical Biomarkers Once PTOA Is Diagnosed

Two studies showed that progression to clinical PTOA can occur as early as 1 year or less following ACL and/or meniscus injury [38,39]. Larsson et al. demonstrated that while SF sGAG, ARGS aggrecan fragments and aggrecan levels were significantly elevated at a mean of 10 days post-injury, only the ARGS aggrecan fragments remained significantly higher in the SF of patients diagnosed with early to late PTOA (mean 1 of year post-injury; range of 3 months to 36 years post-injury) vs. healthy controls [38]. Panina et al. showed that, compared to healthy controls, progression to clinical PTOA can occur as early as 1 year or less following meniscus injury [39]. Patients diagnosed with either early- (Kellgren–Lawrence grade 1–2) or late-stage (grade 3–4) PTOA had significantly higher levels of IL-6, NO and uric acid in the plasma, while plasma IL-1β was significantly increased only in the early-stage PTOA group. Leptin, a hormone associated with obesity that is known to play a catabolic role in articular cartilage, especially in women [62], was significantly increased in the late-stage PTOA group compared to the control group. Cytokines in the SF were not vs. controls and no significant differences between early-stage vs. late-stage PTOA were detected. However, correlation analyses showed that the levels of NO, IL-6, and IL-18 in the plasma significantly correlated with those in SF. SF and plasma leptin levels and SF IL-18 significantly correlated with PTOA severity and PTOA progression. Moreover, there was a 1000-fold increase in IL-6 in the SF compared to the plasma and the plasma IL-6 concentrations were significantly higher than healthy controls, interestingly, there was not a significant increase in SF or plasma TNF-α levels compared to controls or between patients with early and late PTOA. Other studies have shown similar results in patients diagnosed with knee OA. Thus, TNF-α was not increased in the SF [55,63] or the serum of patients diagnosed with knee OA [64]. However, the data suggest that both IL-6 and TNF-α chronically persist years after knee trauma (Figure 1).

Similar to the results from Ersoy et al. [39], another study showed that the serum nitrate and nitrite levels, measured by the same assay, in 36 OA patients were significantly higher than in 30 healthy controls [65]. Other OA studies have corroborated increased uric acid levels in subjects with knee OA. Serum uric acid levels predicted future joint space narrowing in knee OA patients [66] and, while the mean serum uric acid concentrations were significantly higher than their paired SF uric acid concentrations, SF uric acid strongly correlated with SF inflammatory cytokines IL-1β and IL-18 [67].

### 3.6. The Role of Inflammation in the Pathogenesis of Knee PTOA—Summary of Clinical Proof

Compiling the available clinical data, we developed a chronological representation of specific inflammatory and other biochemical biomarkers that significantly increase following knee joint trauma (Figure 1). It is clear from the clinical studies discussed above that knee joint injuries lead to a normal acute inflammatory response following the initial trauma and, importantly, this is followed by a continuous phase of inflammation lasting from months to years following knee trauma, indicating the importance of the early regulation of the immune response for potential clinical benefit. Load-bearing macromolecules of the articular cartilage ECM (notably, ARGS aggrecan and COMP fragments and sGAG), followed by collagen cleavage products, become clinically measurable in the SF early after knee joint injury and continue to be present, signifying early and possibly even irreversible damage to articular cartilage. Not surprisingly, there is a high patient variability between individual, but local joint inflammation often persists in what we defined as the chronic phase of inflammation characterized by higher-than-normal levels of inflammatory cytokines IL-6, TNF-α and IL-17 in the SF. Evidence suggests a systemic response from 1 weeks to 5 years after knee injury, as specific markers are detectable in the contralateral uninjured knee, serum and urine. Once PTOA is clinically diagnosed, there is a systemic inflammatory response with patients exhibiting higher than normal levels of IL-6, IL-1β, leptin, NO, uric acid, ARGS aggercan fragments and possibly complement pathway activation products in the SF and/or plasma.

## 4. Results of the In Vivo, Ex Vivo and In Vitro Models That Simulate Inflammation and/or Injury of the Knee Joint

### 4.1. In Vivo Knee Joint Inflammatory, Injury and PTOA Animal Models

In vivo models are useful for mimicking clinically relevant injury conditions and help us to understand the factors that contribute to knee PTOA disease onset and progression. The majority of knee injuries included in the clinical part of this review were ACL/MCL injuries or articular fractures (Table 2). Several clinical studies also reported rapidly developing hemarthrosis in the acutely injured knee [23,36,68]. We will briefly discuss selected models that mimic these clinical scenarios and show that they induce, to various degrees, subsequent structural damage, inflammation, and/or biomechanical changes that eventually lead to the degeneration of articular cartilage and knee PTOA.

Several studies have shown that intra-articular injection of the cytokines TNF-α, IL-1β, or IL-17 alone (i.e., without applying injury) [69,70,71] are capable of producing some of the injurious effects observed in the different acute, subacute and chronic phases after clinical trauma. Sixteen hours after the intra-articular injection of 10 µg of TNF-α into rat knee joints, a maximal aggrecan loss was observed that was followed by gradual recovery of the proteoglycans in the articular cartilage after 72 h [71]. Likewise, the levels of sGAG in the SF significantly increased vs. control between 8 and 16 hours after TNF-α treatment, but returned to normal levels within 24–48 h suggesting that TNF-α alone may not induce a profound effect on ECM degradation. A single intra-articular injection of IL-17 caused NO release into the SF in rats, similar to IL-1β, IL-17 also dose-dependently inhibited proteoglycan synthesis, but to a lesser extent than IL-1β [69,70]. IL-17 also upregulated the expression of IL-1β. Moreover, three intra-articular injections of IL-17 (1, 10 and 50 ng/mL) for up to 6 days in New Zealand rabbits led to an early significant increase, at 72 h after the last injection, in the expression of MMP-1, -3, and -13, as well as ADAMTS-4 and -5 and COMP, along with the chondrogenic markers COL10A1 and COL1A2; and, concomitantly, a significant decrease in COL2A1 expression [70]. With the exception of MMP-13, which decreased at 12 weeks post-injury, the expression of these markers increased over time until 12 weeks after injection. Furthermore, at the later time points (3, 6 and 12 weeks) the intra-articular injection of the higher doses of IL-17 progressively caused cartilage defects and an increase in the synovial thickness and synovium cell number. Interestingly, the same study showed that the intra-articular injection of IL-17 was capable of inducing PTOA similar to rabbits having their anterior and posterior cruciate ligaments transected and the medial meniscus excised. Likewise, the levels of sGAG in synovial fluid significantly increased vs. control between 8 and 16 h but returned to normal levels within 24–48 h. Interestingly, in antigen-induced experimental arthritis, IL-6 has been shown to function upstream of IL-17, but has also been shown to be a downstream target of IL-17A, and the combination of IL-6 and IL-17A synergistically generated a positive IL-6 expression feedback loop that resulted in excessive IL-6 signaling through the soluble IL-6 receptor (IL-6R) signaling pathway [72]. These data collectively suggest that, while trauma is the initiating factor of inflammation in the clinical setting, pro-inflammatory cytokines alone, and particularly IL-17, can replicate injurious effects.

Joint bleeding is very common after acute knee injury and occurs in up to 98% of knee injuries [23,28,36,68,73]. The blood entering the joint generally reaches up to 100% volume/volume (*v*/*v*) and is cleared within a week if the bleeding stops [73]. When joint bleeding is prevented during surgery, the severity of synovitis and the infiltration of mononuclear cells significantly decreases [74]. Only one study has mimicked acute joint bleeds of the knee joint in animals by injecting (20% *v*/*v*) of coagulated blood for up to 4 days. This canine blood-induced injury model increased synovial inflammation (OARSI score) and decreased sGAG content for up to 4 weeks [73]. The exposure of healthy cartilage tissue to a minimal amount of blood (only 10% *v*/*v* blood) for two days was capable of increasing general MMP activity and long-term articular cartilage damage measured by decreased proteoglycan synthesis and content [75]. In other studies, exposure of healthy human cartilage to 50% *v*/*v* blood similarly decreased proteoglycan synthesis, increased sGAG and resulted in temporal release of IL-β, TNF-α, and IL-6 for up to 10 days [76,77,78]. Traumatized (i.e., bluntly injured) OA articular cartilage explants exposed to (20–30% *v*/*v*) human serum and also cartilage homogenate caused significantly more deposition of complement C5b-9 (i.e., TCC), increased the expression of several injury/inflammatory genes, including MMP-13, IL-8, and CXCL1, and increased chondrocyte cell death vs. trauma alone [52]. Moreover, the inactivation of complement by heat inactivation of the serum prevented most of the effects, suggesting that the exposure of cartilage tissue to blood potentiates injurious effects through complement system activation. In the human arthritic joint, complement proteins are produced by synoviocytes, chondrocytes, macrophages and osteoblasts in the subchondral bone and the activation of complement increases in response to IL-1β, TNF-α or blunt mechanical injury in the presence of serum [52,79,80,81,82,83,84,85,86]. While the evidence to date is inconclusive on whether this could account for the increased complement components in the SF in the acute stage of inflammation after knee trauma (Figure 1), it suggests that such models may be useful for understanding the early acute to subacute blood-induced injurious mechanistic effects of knee trauma.

Furman et al., in 2007, were the first to report a non-invasive intra-articular tibial plateau fracture PTOA model [87]. This intra-articular high energy fracture model uses a blunt impact to the proximal tibia and has been generally performed in C57BL/6 mice. It is capable of producing a fracture to the articular cartilage and subchondral bone, as well as the release of blood and bone marrow into the synovium, which is accompanied by subsequent synovial inflammation and articular cartilage degeneration 2 to 52 weeks after impact [87,88,89,90]. Tibial plateau fracture mouse models using either low or high energies since then have shown similar outcomes with the loss of articular cartilage and sGAG depletion, reduced chondrocyte viability, synovitis, and the presence of circulating IL-1α, IL-β, TNF-α, and IL-6 [89,90,91] demonstrating that such fracture models mirror all stages of clinical PTOA. Moreover, it has a very high (87–95%) success rate in creating a fracture [92]. Similar to articular cartilage injury models that show that mechanical force cause various types of damage ranging from single cartilage ECM fracture to full intra-articular fractures [93,94,95], this type of intra-articular fracture injury model is capable of resulting in both simple and complex fractures of the tibia and, thus, resembles sports and accident-induced high impact joint injuries.

It has been shown that injuries of the ACL and menisci result in PTOA ≤1 year [38,39] or up to 10 to 20 years after initial injury [3]. Commonly used models for ACL injury include non-invasive and invasive models. A non-invasive ACL rupture single tibial compression overload model applies a single load of 8–12 N at both high (500 mm/s) [96] and low speeds (1 mm/s) in C57BL/6 mice [96,97]. While the two injury models did not significantly differ from one another with respect to long-term changes in bone structure, joint laxity, and articular cartilage degeneration, the models were able to induce moderate to severe PTOA 8–10 weeks post injury. Low speed injury also caused synovial hyperplasia (days 3–15), synovial inflammation (days 3–28) as well as sGAG loss (8 weeks) and fibrosis (24 h–8 weeks). Using an in vivo fluorescence reflectance imaging (FRI) quantification method, this same group demonstrated that the high speed injury model increased the activity of cathepsin proteases produced by activated macrophages and neutrophils, as well as the activity of MMPs and cathepsin K, which is involved in bone resorption and aggrecan degradation and, interestingly, while the levels peaked 1–7 days post-injury, their activity remained present up to 8 weeks post-injury [97], further confirming the chronic presence of activated macrophages and neutrophils after knee trauma. Another study showed that three different compression forces (3, 6, and 9 N) applied for 60 cycles with 10 s of rest between each cycle similarly increased the apoptosis of chondrocytes, COL1 expression (day 14), cartilage matrix degradation, and the disruption of the cartilage collagen fibril arrangement and decreased the pericellular aggrecan intensity in the injured region, while the high energy injury caused faster and more substantial post-traumatic synovitis and fibrotic scores, cell death in the deeper zone cartilage, and loss of proteoglycans compared to moderate and low energy injuries [98]. All of the compressive (low to high) forces also mimicked the systemic effects of injury as a similar, albeit lower, concentration of these markers was present in the uninjured contralateral limb and COMP fragments were present in the serum. Trabecular bone loss also occurred in the uninjured contralateral limb. Because this model ruptures the ACL by mechanical forces that are externally applied to the joint, this may also result in injury to other joint structures (i.e., cartilage, menisci, and subchondral bone), depending on the amount of force applied. This model is easy to perform, highly reproducible, and avoids confounding factors, such as infection, that may be associated with invasive injury models and is representative of the clinical phases of PTOA. However, it needs to be taken into consideration that the murine model is better for investigating the acute to subacute phases of injury, as it results in severe posterior bone erosion on the medial side of the tibial plateau, which is reportedly atypical of ACL injury-induced PTOA in other animal species or in humans [96]. It also often causes extreme erosion of both bone and cartilage 10 or more weeks after injury.

Other animal studies included invasive injury models by applying ACL surgical transection (ACLT) with or without additional partial medial meniscectomy (MMX) to investigate the impact on articular cartilage and the expression profile of various inflammatory and chondrogenic markers. Bajpayee et al. performed unilateral ACLT on 3-month-old mature female New Zealand rabbits and found that, compared to the contralateral uninjured knee, the levels of IL-1β, MMP-1, 3 and 13 significantly increased in cartilage explants 3 weeks after surgery, while ACAN expression levels significantly decreased and stayed significantly low at 9 weeks after surgery. While the expressed levels of MMP-3 and -13 remained elevated in the cartilage samples at week 9 after injury, IL-1β levels significantly decreased [99]. This is in line with the clinical data (Figure 1) showing an initial increase of IL-1β in the acute and subacute phase (up to 1.5 months) after injury which disappears in the chronic phase. In another study, Pickarski et al. compared the effects proceeding two different PTOA models using ACLT and ACLT + MMX in 10-week-old male rats. They demonstrated that, in both models, the expression of MMP-13, as well as aggrecanase-1, significantly increased in the articular cartilage samples at the first time point 1 week after surgery, where MMP-13 continued to increase, reaching the peak at 10 weeks, while aggrecanase-1 levels remained steady but high vs. the contralateral uninjured knee [100]. The gene expression of SOX9 and COL2A, which are chondrogenic phenotype markers of the chondrocytes, increased in the first week but then returned to normal levels by the tenth week after injury and, unexpectedly, the levels of expressed SOX9 and COL2A were lower in the ACLT + MMX model, compared to the ACLT model. However, both ACLT and ACLT + MMX models lead to degradation of the articular cartilage. It is notable that this invasive ACLT model may lead to unintended outcomes due to the disruption of the natural joint environment, e.g., incomplete retinaculum repair with patella maltracking, which may increase the rate of subsequent joint degeneration [101]. This procedure may also irritate the fat pad that could additionally increase intra-articular inflammation. Therefore, it has been suggested that this model is not entirely representative of human ACL injury.

When considering the use of animal models, it is important to note genetic and/or anatomical differences in animals compared to humans [102,103,104,105,106]. The injury models discussed here have been performed on mice, rats and rabbits. Each of these animal species has their advantages and disadvantages [102,103]. While naturally occurring OA is common in rabbits, they have a much higher chondrocyte density than other species, and the thickness and cellularity of the transitional and radial zones of chondrocytes is highly variable. Moreover, rabbit articular cartilage spontaneous heals and regenerates, especially in young animals up to 5 months of age. Rodent models have a significantly smaller joint size and thinner articular cartilage than humans, but they serve as the most cost-effective models for feasibility, preclinical studies and mechanistic studies. Mice do not exhibit distinct chondrocyte zonal depth arrangements as humans do in the superficial, transitional, and radial zones of the cartilage. Additionally, the radial zone makes up nearly two-thirds of the rat articular cartilage thickness. Another microstructural difference is that human articular cartilage displays joint surface-specific cell arrangements in the large joints termed “superficial chondrocyte spatial organization”, which other species do not display [107,108,109,110]. In the context of inflammation and articular cartilage regeneration, it is important to point out that certain mouse strains (e.g., MRL/MpJ, LG/J, and LGXSM-6 mouse strains) have shown a superior regenerative and healing response of the articular cartilage, as well as other tissues, compared to other mouse strains (e.g., C57BL/6, LGXSM-33) [104,105,111,112]. In fact, in a closed articular tibial plateau fracture, the PTOA model MRL/MpJ mice had lower systemic IL-1α and higher anti-inflammatory cytokines IL-4 and IL-10 present than C57BL/6 mice. When comparing the injured to the uninjured knee, in contrast to C57BL/6 mice, MRL/MpJ mice showed no differences in the histologic grading of articular cartilage degeneration, bone density, or subchondral bone thickness [111]. This suggests that, e.g., C57BL/6 may be more beneficial for knee PTOA studies. Moreover, unlike rats, mice have naturally occurring OA as well as a rapid disease onset. Importantly, the availability of genetically modified mouse strains provides the opportunity to study the molecular mechanisms contributing to PTOA development.

### 4.2. Considerations in Using In Vitro Chondrocyte and Ex Vivo Articular Cartilage Models

While the experimental environment of cell-based or articular cartilage explant models may not fully simulate the joint tissues post-trauma, such models decrease the overall complexity and allow application of injury and/or inflammatory cytokines in controlled, defined microenvironments. Ex vivo tissue articular cartilage explant studies permit the preservation of the cells in their natural three-dimensional environment and the maintenance of cell–matrix interactions and cartilage zonal cell arrangements. Moreover, mechanical injury, e.g., through single vs. repetitive impact, can simulate different types of trauma or ranges of trauma extent and, thus, apply different forces to the tissue and the cells within their native ECM. Such studies allow us to probe input–output behavior and bridge gaps in mechanisms of post-injurious inflammation, helping to identify the specific mechanisms that contribute to post-injurious perpetuation of inflammation and subsequent PTOA. These studies also allow for linking the observed effects to both cell and tissue behavior, helping us to understand cytokine and/or cell–cell crosstalk, and will one day help in explaining the presence of differing responses between patient groups and the progression to PTOA in many, but not all, patients.

Before we discuss these models, we will discuss the source of articular cartilage tissue used in in vitro and ex vivo models. The majority of cell-based in vitro studies have used primary chondrocytes of human origin. Others have used primary bovine chondrocytes or cell lines, such as the chondrosarcoma SW-1353 human cell line, the murine chondroprogenitor ATDC5 cell line derived from AT805 teratocarcinoma cells or immortalized human juvenile costal-derived chondrocyte T/C-28a2 cells. Obviously, there are pros and cons for each source. Cell lines are convenient, but they do not fully represent a primary cell. For example, T/C-28a2 and SW-1353 human cell lines are not suitable for studying NO release and iNOS expression because, in comparison to ATDC-5 cells, these cells are incapable of producing NO and expressing iNOS in response to LPS, IL-1α, or IL-1β treatment [113]. ATDC5 cells are an acceptable alternative as they imitate the articular chondrocyte phenotype and they have been successfully used in inflammatory in vitro models of OA [113,114,115,116,117]. However, an important question remains; namely, how the accumulated data translate to the clinical outcome.

Articular cartilage explant studies tend use bovine or human tissues. The advantage of using bovine tissue is that it is easily accessible and a healthy source of young and adult tissue that could be used to better understand disease progression in different age groups, especially if both injury and inflammatory factors are applied. Another advantage of bovine cartilage explant is the development of naturally occurring OA in multiple bovine joints that very closely mimic the onset and progression in aging humans [103]. Therefore, bovine tissue is useful for investigating the early disease onset or in preclinical assessments aimed at prevention of early disease. One disadvantage of bovine cartilage is its decreased thickness and higher cellularity compared to human cartilage [100]. However, access to healthy human cartilage is difficult and, for this reason, most of the human injury and/or inflammatory studies have been performed on tissue explants or on chondrocytes isolated from human patient OA articular cartilage. While some studies grade their tissue and use “healthier” parts, e.g., macroscopically intact-appearing areas, the tissue cannot be considered truly healthy due to disease presence and exposure to inflammatory cytokines and degradative enzymes present in the joint. However, the use of different grades of diseased OA tissue may be beneficial for simulating chronic inflammation, especially if the media is supplemented with the chronic inflammatory cytokines present in the diseased joint (Figure 1). This suggests that, in some settings, human tissue-based models may be more relevant due to the morphological, physiological and biochemical tissue properties, whereas basic science studies can very well address mechanistic questions in cells and tissues other than human. Because cells and tissues are derived from patients with differences in, e.g., age, genetics, or co-morbidities, it is often unavoidable to investigate a larger number of patient tissue explants or use stratification strategies to better understand outcomes and treatment effects.

An important consideration of inflammatory or other in vitro studies is the arising dedifferentiation of chondrocytes that stem from culturing cells on conventional tissue culture plastic [118]. It is well known that chondrocytes in monolayer culture undergo in vitro dedifferentiation exhibited by a change in their cell morphology, transitioning from a spherical morphology to a flat spindle-like morphology, with a corresponding change in the cell gene expression profile exhibited by an increase in type I collagen (COLIA2) and decrease in type II collagen (COL2A1) expression that results in lower quality cartilage ECM. This process occurs early after the expansion of primary chondrocytes in cell culture and increases with increasing passage [119,120]. For example, previous studies have used alginate bead and pellet culture [121,122], serum or growth factors combined with subsequent alginate bead cultures [123], pellet cultures combined with low oxygen concentrations [124], co-cultures with mesenchymal stromal cells (MSCs) on a porous surface [125], agarose hydrogels with varying RGD adhesion site densities and mechanical properties [126], single-component photo-crosslinkable hydrogels [127], and chimeric Activin A/BMP2 ligand AB235 [128] to attempt re-differentiation of passaged chondrocytes. These studies generated valuable insight by inducing the re-differentiation of human OA chondrocytes obtained during joint replacement procedures [121,123,124,127,128] and of porcine and bovine chondrocytes [125,126]. In one of our own studies, we cultured patient- and joint surface-matched human OA chondrocytes vs. OA chondrons, which are OA chondrocytes within their retained pericellular matrix, in a self-assembling peptide hydrogel. Interestingly, human OA chondrons displayed a significant long-term survival advantage over chondrocytes in hydrogel culture [129]. Thus, retaining a native cartilage structure presented a significant advantage in stabilizing the chondrocyte phenotype. In another study, we evaluated human chondrocytes isolated from articular cartilage lesion sites vs. knee joint notch sites and showed that, after monolayer expansion, re-differentiating lesion chondrocytes cultured in alginate beads resulted in a pool of cells with a greater chondrogenic potential compared to expanded and de-differentiated notch chondrocytes, indicating that ex vivo re-differentiated lesion chondrocytes may hold non-utilized clinical potential for tissue engineering articular cartilage [130]. On a side note, such location-based investigations are important because chondrocyte metabolic characteristics differ across joints [131,132,133,134,135], whereas other characteristics such as the abovementioned chondrocyte arrangements within the tissue differ, even between individual joint surfaces within the same joint [107,110] and between different stages of OA [108,109,136]. Thus, while there are various methods to delay chondrocyte dedifferentiation, current methods have not achieved complete success in preventing this process. However, because relatively few chondrocytes are present in the tissue, the expansion of chondrocytes is essential to achieve experimentally (and clinically) relevant cell numbers. Therefore, although these studies are essential, these points need to be considered.

A study by Tsuchida et al. elucidated the effect of culturing chondrocytes on the production of various inflammatory mediators [137]. This study showed that most of the concentrations of inflammatory cytokines, such as IL-1α, IL-6, and TNF-α in the cartilage from tissues with grade III and IV focal defects or from OA tissue were 10- to 100-fold higher than those measured in the SF. Remarkably, the cytokine production by chondrocytes in cell culture was much higher for many inflammatory mediators, compared to articular cartilage. This is in contrast with the general belief that synovial cells are the main source of inflammatory mediators in articular joints and, instead, indicate that the chondrocytes within their native tissue can act as a primary source of the production of many cytokines. Moreover, there were large variations in the concentrations of these inflammatory mediators after 7 days of culture of chondrocytes, which were seeded onto collagen-coated plates directly after isolation vs. those that were seeded at passage 2. In addition, some cytokines, such the pro-inflammatory IL-1α and IL-8 cytokines and anti-inflammatory IL-4, IL-10 and IL-13 cytokines, were absent in protein extracts from healthy native tissue, whereas they were produced in healthy chondrocyte cultures. This suggests a response to culture, although a head-to-head comparison of directly isolated chondrocytes and cultured chondrocytes vs. isolated cartilage explants, as well as cultured cartilage explants would be needed to fully conclude this. These results and the resuls of another study [138] show that chondrocytes are clearly capable of contributing to inflammation, and this process may represent a normal healing or regenerative response to cell culture. These findings should be considered in future in vitro studies using chondrocytes as a representative model by clearly stating and discussing the cell source and culture details to avoid misleading data and/or inaccurate interpretations. This also further supports performing combined studies at multiple levels when assessing inflammation or anti-inflammatory treatment strategies for a better representation of the full narrative.

### 4.3. In Vitro Chondrocyte and Ex Vivo Articular Cartilage Explant Injury and/or Inflammatory Models

Other reviews [139,140] have well described how articular cartilage explant models have used various types of injurious compression protocols to simulate mechanical injury and have demonstrated that, despite differences in protocols, these models show that they similarly and successfully induce the clinically observed pathologies following trauma to the knee joint. Hence, the mechanically-induced injury of articular cartilage leads to a significant increase in chondrocyte death, ECM loss, expression of matrix-degrading enzymes and a decrease in the COL2 gene and, to some extent, an increase in inflammatory cytokines even in the absence of inflammatory cells, such as macrophages [141,142,143,144,145,146,147,148,149,150,151,152,153]. Some studies have used a combination of mechanical injury and inflammatory cytokine treatment, typically using IL-1α, IL-1β, TNF-α, IL-17, or the combination of TNF-α/IL-6/sIL-6R [143,144,145,146,148,152,154] to mirror trauma and the presence of cytokines that are significantly present with the injured knee. 

Articular cartilage explant and in vitro chondrocyte studies, using cells or tissue derived from either OA or healthy sources have shown that IL-1α treatment alone is capable of increasing production of MMP-1, 3, 9 and -13 and ADAMTS-4 and -5, decreasing proteoglycan synthesis and increasing the loss of sGAG [144,148,149,155,156,157], while IL-1β alone is capable of enhancing NO production, collagenase activity, IL-6, IL-8, LIF, MMPs (MMP-1, -3, -13, -7, -9, -12), complement components, chemokines as well as aggrecan release; and inhibiting type II collagen, NF-ĸB binding activation and phosphor-Smad2/3 [69,147,152,158,159,160,161,162,163,164]. These studies, as well as animal models, showed that IL-1β is involved in inflammation and cartilage degradation. However, Bougault et al. demonstrated that IL-1β was not produced by OA articular cartilage itself but rather by synovial tissue [165]; therefore, the source of IL-1β could be synovial fibroblasts or other cells such as macrophages.

TNF-α alone similarly increased IL-6, IL-8 and LIF, MMPs (MMP-1, -3, -9 and -13), ADAMTS-5 and -4, complement components, chemokines as well as aggrecan fragment and sGAG release and decreased COL2 expression of isolated chondrocytes but only increased NO production when used at high concentrations [143,144,145,146,147,157,160,161,166,167,168,169]. In addition, TNF-α induced the expression of IL-1β in healthy human chondrocytes [166]. Moreover, Patwari et al. showed that the combination of injury and TNF-α caused higher loss of sGAG compared to either alone [144].

IL-17 alone dose-dependently increased MMP-1, -3, -13, and MCP-1 and decreased TIMP, COL2A1, and SOX9 mRNA expression in human OA chondrocytes [170]. When IL-17 was combined with low concentrations of IL-1β, the combination increased NO production and decreased GAG synthesis in a rat patellar articular cartilage explant model greater than either cytokine treatment alone. This study, like other studies on articular cartilage explants, chondrocytes and/or synoviocytes suggest that IL-17 acts synergistically with IL-1β or TNF-α [152,153,169,171,172,173,174,175,176,177,178], potentiates inflammatory gene expression (e.g., IL-6, IL-8, TNF-α) and MMP expression, and causes catabolic effects, such as ECM component loss and a decrease in the mRNA expression of chondrogenic markers, such as SOX9, XYLT1, COL2A1, and ACAN. IL-17A also caused the release of MMP-2, and -9, sGAG, the ADAMTS-mediated aggrecan degradation fragment (exAGNx1) and MMP-mediated type II collagen (C2M) in a bovine full-depth articular cartilage [150]. Moreover, this study showed that the proteomic analysis of conditioned media from IL-17A treated articular cartilage revealed an upregulation of IL-6, MMP-3, ADAMTS-4, neutrophil/macrophage chemoattractants CXCL6 and CCL20 (also known as macrophage inflammatory protein-3 alpha, MIP-3α), complement factor B, latent TGF-β-binding protein 2 and chitinase 3-like 1 (CHI3L1 or YKL-40), a secreted glycoprotein linked to OA, compared to control-treated tissue. This study and another study suggest that the exposure of chondrocytes and synoviocytes to IL-17, which led to the increased expression of granulocyte-attracting regulating genes and release of chemokines, could further drive inflammation by inducing the influx of mononuclear cell influx, such as macrophages [173,174].

IL-17 also inhibited TGF-β3-induced the chondrogenic differentiation of human MSCs [179], which may be locally present after knee trauma, or at least after surgical procedures, such as microfracture [180] or combined microfracture and autologous chondrocyte implantation (ACI) [181,182]. Therefore, theoretically, TGF-β would also be subjected to IL-17 modulation. For other effects of TGF-β on chondrocytes and/or MSCs, we refer the interested reader to our recent reviews [13,183].

Many IL-17 effects can be reversed by the removal of e.g., IL1-β and IL-17A from the media [152] or by pre-treatment with an IL-17 receptor antibody [175], dexamethasone, or kinase inhibitors [172,176]. Secukinumab, a human monoclonal antibody that binds to IL-17A, is already used clinically to treat arthritis-associated symptoms and joint disease damage in ankylosing spondylitis and psoriatic arthritis, further supporting the antagonistic role of IL-17A in joint disease [184]. These studies demonstrate that, like TNF-α or IL-β, IL-17 is strongly capable of enhancing inflammation and causing catabolic effects that lead to degeneration of articular cartilage; and that potentiating effects of cytokine combinations with IL-17 surpass those of individual cytokines. This review, in the context of PTOA of the knee, as well as others reviews, suggest that IL-17 is a crucial pathogenic molecule in local and systemic disease pathogenesis in many types of disease tissues and that IL-17 signaling may promote chronic disease development [185,186,187,188,189] emphasizing the need for further mechanistic IL-17 studies in various contexts.

Whereas most studies generally focus on the use of one cytokine, a few studies have used a combination of TNF-α, IL-6 and soluble IL-6 receptor (sIL-6R) to better simulate the inflammatory environment existing after knee trauma. Before proceeding to their effects on articular cartilage, we first briefly discuss the two mechanisms, by which IL-6 interacts with target cells [190,191]. IL-6 is capable of binding to the membrane-bound IL-6 receptor (IL-6R), a receptor that alone lacks signaling capacity because it does not contain a signal transduction domain. When the complex of IL-6/IL-6R binds to a second membrane receptor called glycol–protein 130 (gp130), the dimerization of gp130 occurs, which initiates intracellular signaling. This “classical” form of IL-6 signaling is important in the acute phase of inflammation. Interestingly, while gp130 is found in all cell types, the membrane-bound IL-6R is expressed in only a few cell types, such as neutrophils, monocytes, and macrophages as well as in naive and memory T cells. IL-6 can also exert its effects through a second mechanism by interacting with the soluble form of the IL-6 receptor (i.e., sIL-6R) that is generated through proteolytic actions (e.g., by protease disintegrin and ADAM enzymes). The sIL-6R binds to IL-6 with a similar binding affinity as the membrane-bound IL-6R and rather than competing with the membrane-bound IL-6R, the complex of sIL-6R/IL-6 bind to gp130-expressing cells and, thereby, induce intracellular “trans-signaling”. This mechanism of signaling leads to a more robust activation of the IL-6 intracellular signaling pathway that promotes the chronification of disease [191].

In healthy bovine articular cartilage, the addition of IL-6 and sIL-6R alone was able to increase the latent form of MMP-3 (pro-MMP-3), with increases in proMMP-3 being more prominent with the addition of TNFα [146]. However, the application of mechanical injury and TNF-α/IL-6/sIL-6R to articular cartilage markedly increased MMP-1, 3, and -10 and aggrecanase-mediated, rather than MMP-mediated, aggrecan catabolism and produced a more pronounced loss of sGAG and COMP and COMP Ser^77^ neoepitopes [143,145,146,192,193]. The exposure of non-injured articular cartilage to TNF-α/IL-6/sIL-6R alone for up to 3 weeks resulted in a similar release of both intact COMP and COMP Ser^77^ neoepitopes into the surrounding media of articular cartilage explants to levels found in the SF of patients suffering from acute knee pain with or even without acute trauma [192]. Therefore, such combinations may be more suitable for understanding the complex, not yet understood chronic effects after injury.

### 4.4. Co-Culture Studies

Even though chondrocytes are a strong source of pro-inflammatory cytokines, as discussed above, the elevated levels of inflammatory cytokines in both articular cartilage and SF suggest the role of other cells, in addition to chondrocytes, in governing inflammation. Therefore, co-culture studies can aid in understanding the role of specific cell types, such as macrophages or synovial fibroblasts, in the development and progression of PTOA.

Several studies demonstrated the importance of synovial fibroblasts as well as macrophages in clinical knee OA [174,194,195,196,197]. Synovial membrane biopsies from untreated PTOA knee joints contain a high number of macrophages and fibroblasts as well as thicker collagen bundles in the sublining and subsynovial regions of the synovium [194]. Wood et al. detected the presence of two types of macrophages in patients, with knee OA having distinct functional gene signatures, which were defined as articular cartilage remodeling and inflammatory macrophages, respectively [196]. The presence of soluble macrophage CD163 and CD14 markers in the SF and plasma were shown to correlate with pain and with OA structural damage progression, i.e., joint space narrowing and osteophyte severity, in the knee joint [195]. After fracture of the tibia plateau MRL/MpJ mice, which are known to generally heal and regenerate tissues faster [104,105,111], had a lower intra-articular and systemic inflammatory response compared to wildtype CD7BL/6 mice which may be attributable to reduced synovial macrophages present after joint injury, as macrophage chemoattractants were also reduced in these mice [112]. Together, these studies suggest that macrophage-related inflammation help drive and may predict PTOA progression.

To resemble the in vivo joint, some studies have used co-cultures of bovine articular cartilage explants combined with the joint capsule containing the synovial membrane [198,199,200], as it is responsible for producing the SF and contains synovial fibroblasts, as well as lymphocytes and monocytes that could affect the inflammatory environment. In these studies, the proteolytic activity of both MMPs and aggreganases were elevated when the synovial joint capsule was added to mechanically injured cartilage, and, consequently, the concentration of released aggrecan fragments, e.g., ARGS, was higher in the medium, while GAG synthesis was significantly decreased, demonstrating the direct contribution of the synovial joint capsule in post-injurious articular cartilage damage. Interestingly, this same group showed that the human and bovine joint capsule from a normal knee joint released a 20–25 kDa heat-labile factor that was capable of causing a 40 to 60% decrease in cartilage proteoglycan synthesis and the inhibition of IL-1 and/or TNF-α was unable to prevent sGAG loss, suggesting that the joint capsule is capable of releasing an IL-1 or TNF-independent pathway cytokine or other factor, which strongly inhibits cartilage biosynthetic activity [200]. IL-17 could be a candidate for this factor, as it is within this molecular weight range.

Similarly, Beekhuizen et al. used a human in vitro single vs. co-culture model comprised of articular cartilage explants and synovial tissue to study the contribution of both in OA tissues [201]. Whereas mono-cultured cartilage released IL-1β, IL-4, IL-7, IL-10, and IL-13, the synovial tissue culture released IL-6, IL-8 and IL-1ra. Most of the cytokines detected in the co-cultured articular cartilage/synovial explants matched with cytokines measured in OA SF, suggesting that both tissues are capable of contributing to an inflammatory environment, but with a differential response. Another study demonstrated that the inflammatory milieu of OA SF inhibited the cell viability of chondrocytes, altered their cell morphology by producing smaller and more globular-like cells that had reduced cell-cell contacts and significantly increased the concentrations of IL-6, IL-8, MCP-1 as well as vascular endothelial growth factor (VEGF) [202]. These studies highlight the use of in vitro co-culture studies in gaining insights into post-traumatic mechanisms as well as their contribution in promoting post-injurious effects and inflammation.

In vitro models have further assessed the role of macrophages to understand how these pro-inflammatory or anti-inflammatory cells could enhance the inflammatory environment and OA progression. In a 3D co-culture model containing either healthy chondrocytes or OA chondrocytes and pro-inflammatory M1 macrophages, there was a simultaneous increase in both matrix degradative enzymes, including MMP-1 and -3 and pro-inflammatory cytokines IL-1β, TNF-α, IL-8, MCP-1 and IFN-γ [203], demonstrating that (lipopolysaccharide (LPS)-stimulated) M1 macrophages are capable of initiating inflammation and inducing cartilage degradation at both an early stage (i.e., prior to OA) in healthy cells, as well as during later stages of OA. In line with this study, another study showed that conditioned media from (IFN-γ + TNF-α stimulated) M1 macrophages upregulated IL-1β, IL-6, MMP-13 and ADAMTS5 and inhibited ACAN and COL2A1 expression in human articular cartilage explants, further endorsing the role of M1 macrophages in disease progression [204]. Together, these results manifest a remarkable effect of the synovial inflammatory environment in the progression of inflammation, articular cartilage destruction and disease progression. All of these studies reinforce the validity of co-culture systems as relevant and helpful PTOA models.

## 5. Clinical Findings vs. Experimental Evidence

### 5.1. Comparing the Concentrations of Inflammatory Cytokines Used in Laboratory Models vs. the Clinical Presentation

While the results of the models discussed in this review match the profile of the clinical presentation of patients after knee joint trauma and, therefore, are useful models for investigating the post-injurious effects and mechanisms of inflammation, it is important to note that most of the laboratory studies applied markedly higher concentrations of IL-1β, TNF-α, IL-6 and IL-17 than those measured in the SF of the knee joint after knee trauma (Table 3), especially IL-1β or TNF-α. While the IL-6 and IL-17 concentrations used in laboratory models were not as exceedingly high, they were still higher than physiological concentrations found after knee joint trauma. Importantly, although this problem in the study design has been raised in the context of OA [185,205], the present review concludes that a comparable problem affects studies pertaining to PTOA. Therefore, future study designs may consider investigating cytokine concentrations that are similar to the range found in the SF of joints after trauma or PTOA. By doing so, one may discover new facets of articular cartilage biology, which is especially important in pre-clinical assessments of acute anti-inflammatory and/or regenerative PTOA treatments aiming to reduce inflammation and early articular cartilage damage.

### 5.2. Comparing the Results of In Vivo, Ex Vivo and In Vitro Laboratory Models vs. the Clinical Presentation

**Figure 2:**
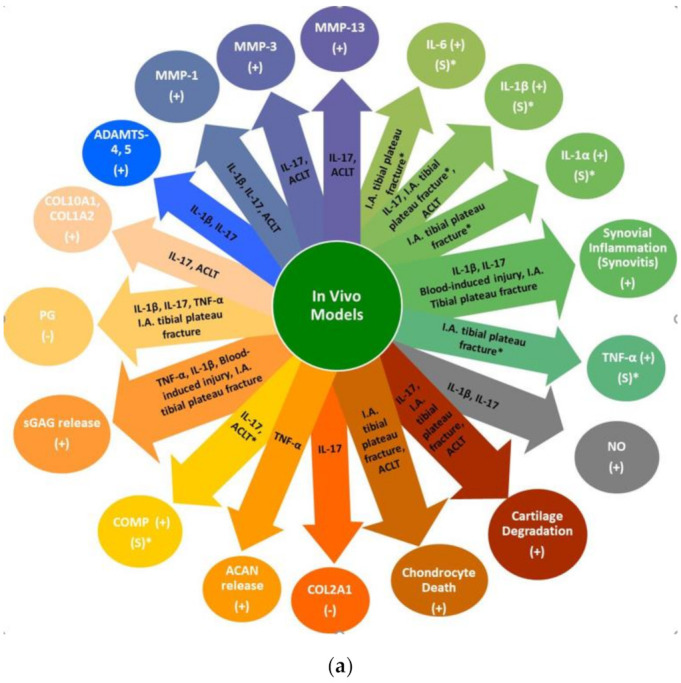

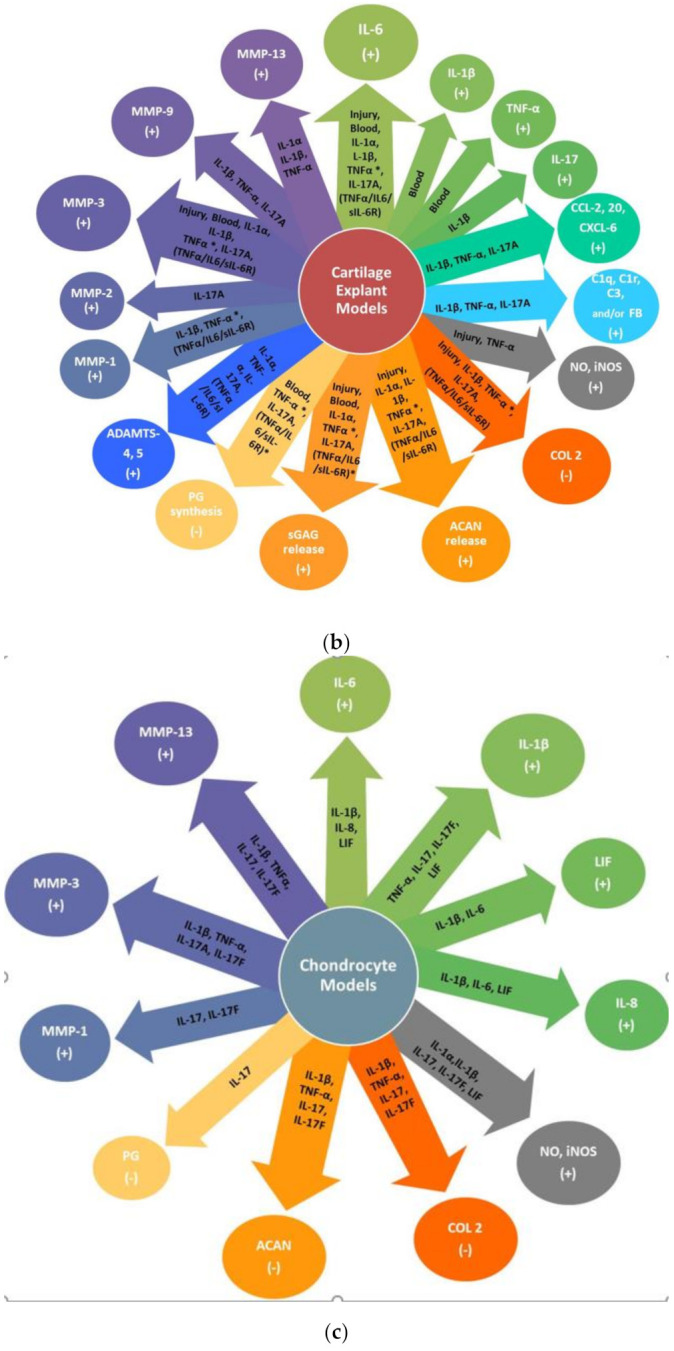
The effects of injury and/or inflammatory cytokines in in vivo models and ex vivo and in vitro models using tissue and chondrocytes from non-0A (healthy) articular cartilage. A (−) sign indicates a decrease and a (+) sign an increase. (**a**) In vivo models effects showing the effects of intra-articular injection of IL-1β [69,70], TNF-α [71], or IL-17 [69,70] alone, blood-induced injury [73], intra-articular (I.A.) acute tibial plateau fracture [87,88,89,90,91,92], or ACLT which includes data from the non-invasive ACL rupture single tibial compression overload model and invasive ACLT model [96,97,98,99,100,101]. The * indicates the corresponding model that showed an increase of that marker in the serum (S). (**b**) Ex vivo cartilage explant models using non-0A (healthy) tissue showing the effects of blood-induced injury [76,77,78], mechanical injury using a single injurious compression without the addition of any pro-inflammatory cytokines [93,141,143,144,206,207], inflammatory cytokines IL-1α alone [144,149,155,156] or combined with injury* [144,148], IL-1β alone [147,152], TNF-α alone [144,145,146,147] or combined with injury* [143,144,145,146], IL-17A alone [150], and TNF-α/IL-6/sIL-6R alone [143,146,154] or combined with injury* [144,148,154]. **(c)** In vitro chondrocyte models using cells isolated from non-OA (healthy) cartilage tissue showing the effects of IL-1α [157], IL-1β [159,160], TNF-α [166,167], IL-17 or IL-17F [172,175], IL-6 or IL-8 [160], and LIF [160,175].

One of the questions that we address in this review is whether the various in vivo, in vitro and ex vivo models of inflammation and/or injury simulate the clinical scenario and adequately replicate the different stages of post-injurious inflammation that occur after acute clinical trauma in the human knee joint. To address this question, much like the clinical studies that generally excluded patients with prior joint pathology, we focused on the animal models (Figure 2A) discussed in this review as well as articular cartilage (Figure 2B) and chondrocytes (Figure 2C) obtained from healthy (i.e., non-OA) tissue.

As shown in Figure 2B,C, the application of blood, mechanical injury, pro-inflammatory cytokines, or their combination in the various in vitro models on healthy chondrocytes and ex vivo models of healthy articular cartilage explants showed similar results to the clinical post-injury data. IL-6, one of the distinctive markers detected in all phases following clinical injury (Figure 1), was significantly increased in these models on the mRNA and protein expression level in response to blood and multiple pro-inflammatory cytokine stimuli (IL-1α, IL-1β, TNF-α, IL-17, IL-8, LIF and the combination of TNF-α/IL-6/sIL-6R) alone. Similarly, as with the increased MMP-3 noted in clinical studies, MMP-3 was increased in vitro and ex vivo in response to injury or to the presence of pro-inflammatory cytokines (IL-1β, TNF-α, IL-17, or the combination of TNF-α, IL-6 and sIL-6R). The breakdown of ECM components (release of aggrecan and sGAG fragments and decreased proteoglycan and COL2 synthesis) was also apparent in response to injury or IL-1α, IL-1β, TNF-α, IL-17, or the combination of TNF-α/IL-6/sIL-6R. Although TNF-α was not measured in articular cartilage/chondrocyte injury and/or inflammatory studies, blood-induced injury models on articular cartilage explants showed that the presence of blood was capable of increasing TNF-α. These data together, with the continued presence of TNF-α in the chronic stage of inflammation after knee joint trauma (Figure 1), suggest that TNF-α is produced by other cell types, such as synovial macrophages, synovial fibroblasts or T helper 17 (Th17) cells.

These data demonstrate that ex vivo/in vitro models using healthy tissue sources are capable of replicating the various clinical phases after knee trauma. When explant models combine injury with the application of pro-inflammatory cytokines, they can simulate the mechanisms and phases of inflammation that are associated with PTOA of the human knee joint. Moreover, Figure 2C confirms that the in vivo models of intra-articular injection of Il-1β or IL-17 or intra-articular tibial plateau fracture or ACL injury recreate PTOA disease progression. Interestingly, the intra-articular tibial plateau fracture model and ACLT models showed that injury resulted in high levels of IL-1β and/or IL-6 in the serum, indicating that both models also successfully mimicked the systemic effect observed in the clinical early and late PTOA stages. Therefore, these models are capable of fully simulating the acute, subacute, and chronic phases of injury and the PTOA of the knee joint.

### 5.3. Assessing Clinical and Experimental Results to Determine if IL-1β, TNF-α, IL-6 and IL-17 Are Possible Causal Factors in Inducing Progression towards Knee PTOA

As in vivo (Figure 2A) and ex vivo articular cartilage (Figure 2B) and chondrocyte (Figure 2C) studies illustrated that the presence of one or more inflammatory cytokines alone (i.e., without application of injury) is equally capable of producing the same effects of those induced by injury, these data confirm that inflammatory cytokines lead to prolonged post-traumatic inflammatory and catabolic effects and function in a damage-perpetuating way similar to injury alone. Because IL-1β, TNF-α, IL-6, and IL-17 were the leading pro-inflammatory cytokines that led to these effects (Figure 2) and predominated in the clinical setting as well (Figure 1), we used the Bradford Hill Framework [19] to establish whether the presence of these factors may lead to a causal PTOA disease effect. As shown in Table 4, nine out of nine criteria were met for TNF-α and IL-6, indicating that there is convincing evidence that the presence of these pro-inflammatory cytokines is causal in PTOA of the knee. Seven out of nine criteria were met for IL-1β and IL-17, indicating that it is credible that these cytokines are causal in PTOA disease progression. The reasons for this credible effect, as opposed to a causal effect, stated for IL-1β and IL-17 will be discussed.

While most of the clinical studies showed that IL-1β was significantly increased vs. healthy controls [21,23,24,25,26,27,29,31,39], some studies demonstrated that IL-1β was not present [28] or that it was only present in a certain percentage of the patients [24,26,29,31]. Evidence from the laboratory models clearly demonstrated that IL-1β leads to post-traumatic PTOA-like effects (Figure 2). There could be several explanations for the lack of detectable IL-1β in some patients, such as common polymorphisms of IL-1β genes that may cause alterations in the transcription of IL-1β that subsequently affects its circulating levels, causing increased the susceptibility to PTOA [208] or, alternatively, a lack of immunoregulators such as IL-1ra or anti-inflammatory IL-10, IL-13 and IL-4 cytokines. Il-1ra was present in the SF after knee trauma in three out of four clinical studies discussed in this review, while one group showed that IL-1ra was significantly lower than the control group 0–48 h after injury and did not increase up to 21 days post-injury [25]. Another study provided insight on this and showed that commonly occurring IL-1ra gene variants, which can post-transcriptionally influence IL-1ra levels [222], associates with radiographic progression of knee OA [223], suggesting that an imbalance of IL-1 may cause disease progression in certain OA cohorts due to a lack of IL-1 control. Moreover, IL-10 [21,24,32,137] was present in the knee joint during acute inflammation, but significantly decreased thereafter. Other anti-inflammatory cytokines, such as IL-4 and IL-13, were also absent [21]. Notably, inflammatory and blood-induced injury models showed that maintaining concentrations of anti-inflammatory cytokines [12,76,77,114,141,142,224,225] or IL-1ra [226,227,228] not only modulated the inflammatory response by reducing inflammation, but additionally stimulated chondro- and cartilage-protective effects. Moreover, IL-10 promoted the regeneration of cartilage tissue [142]. Therefore, while it is not clear whether IL-1β polymorphisms, an imbalance between IL-1 and IL-1ra, or a lack of control of anti-inflammatory cytokines in the early stages after knee trauma, or even other reasons beyond the scope of this review, are responsible for why some patients have excessively high IL-1β while others do not; how this relates to clinical PTOA of the knee remains unclear.

Whereas levels of IL-1β, TNF-α and IL-6 were frequently measured after knee trauma, IL-17 was not. There are two clinical studies that measured IL-17 and demonstrated that IL-17 was present in the acute [21] and chronic phase (3+ months) after injury [33]. However, in [21], IL-17 correlated with macrophage inflammatory protein-1 beta (MIP-1β, also known as CCL4) 24 h after injury and IFN-γ, TNF-α, IL-12, and IL-1ra 24 h and 9.5 days post-injury, whereas IL-17A was not significantly elevated in the injured knees, compared to the controls. Strong correlations were also shown, at both time points, between IL-8 and IL-6 and between IL-1β and IL-2, which negatively regulates Th17 cells, suggesting that Th17 cells could partake in the inflammatory processes associated with injury. In [33], IL-17 was significantly increased in both the serum and SF compared to controls. However, it was not clear exactly how long IL-17 was chronically present, as the authors only reported that fracture patients endured pain for at least 3 months. Therefore, longitudinal studies are needed to relate the IL-17 changes in the SF and serum to the development of PTOA of the knee.

## 6. Summary, Outlook and Early Disease Considerations

The objective of this review was to focus on the current state of the research findings of one joint, the post-traumatic knee joint, after severe acute knee trauma in order to show how inflammation plays a central role not only in the initial post-traumatic pathology, but also in chronic and systemic inflammation, and how it could potentiate PTOA disease. From the clinical studies discussed in this review, it is evident that inflammation plays a key role in both the initial phase after injury and in perpetuating post-injurious pathology towards clinical knee PTOA. This comprehensive review presents, for the first time, a timeline following the initial event of a knee trauma, in which specific inflammatory as well as other biochemical biomarkers, increase in the acute, subacute, chronic and early to late PTOA stages of disease (summarized in Figure 1) after knee trauma. This suggests that specific markers may be used as potential prognostic indicators of disease (e.g., MMP-3, ARGS or COMP fragments, IL-17) and could be used to assess whether a patient is or is not progressing towards the development of clinical PTOA, especially since some of these biomarkers are increased and measurable in serum/plasma.

However, while this review and other reviews and consensus reports suggest that such biochemical biomarkers could be used as indicators of PTOA progression [229,230,231], so far adequate validation, e.g., of MMP-3, is still lacking. As we show in this review, while MMP-3 as well as other biochemical biomarkers remain present for extensive periods of time in the SF after injury, and SF MMP-3 levels even correlated with IL-6 levels in the SF up to 7 months after knee injury [31], SF analysis is not always clinically feasible or SF is not reliably gained. Therefore, measuring suitable biochemical markers in the serum would prove to be more advantageous. However, studies have shown mixed results in terms of correlations between serum MMP-3 levels and clinical signs of synovitis (radiographic or MRI grading) or the grade of articular cartilage damage (arthroscopic grading) [61,232,233,234]. This suggests that a single marker alone or combined with these clinically established articular cartilage-focused imaging or grading methods may not be sufficient. An alternative approach is to use multi-modality measurements with predictors known to be associated with PTOA. The combination of clinical evaluation, radiographic OA grade and ultrasonographic indicators of inflammation (swelling/knee effusion), as well as pain intensity and disease duration, have been used to predict knee joint replacement surgery within 3 years after the initial patient visit [235]. Multivariable analysis has also predicted PTOA [236]. However, these predicting methods predict late-stage changes leading to joint replacement, but not the early processes leading to potentially reversible tissue damage. Moreover, successfully predicting PTOA within 2 to 3 years in patients after meniscus repair or partial meniscectomy after ACL tear is somewhat expectable, as advanced joint deterioration is known to be associated with both procedures [237,238]. Despite these facts, the impressive aspect is that they have successfully demonstrated that multi-modality measurements could be used for prediction.

Another approach is to focus on other diagnostic imaging techniques to diagnose the early and potentially reversible disease pathology, especially since methods, such as X-ray and MRI cannot yet statistically discriminate between healthy vs. early OA [239]. We recently showed that a clinically available confocal laser endomicroscope could be used to image a surrogate marker of otherwise clinically, but not yet detectable, early OA functional pathology prior to cell clustering [240]. Specifically, we showed that characteristics of the spatial organization of the superficial zone chondrocytes (SCSO) are indicative of early pathology [94,107,108,109,110,136,241] and can be imaged clinically using a confocal laser endomicroscope. Combined with random forest modelling, this artificial intelligence (AI)-based non-destructive quantitative optical biopsy was capable of accurately differentiating between a healthy vs. early OA architectural fingerprint, suggesting that early OA pathology detection is indeed possible with current clinical technology [240]. Thus, such new imaging approaches, combined with modern analysis and/or predictive tools, such as AI, could be used with measurements of multiple easily measurable biochemical biomarker candidates (e.g., MMP-3 and possibly IL-17) from blood samples and combined with patient-related parameters over time and in parallel. Using such an integrated approach could facilitate the detection of earlier pathological and functional changes compared to current techniques. In this context, a multi-disciplinary international group of experts has published guidelines on the design and conduct of interventional studies aimed at preventing PTOA following acute knee injury [229], which are not only relevant to assessing treatment outcomes but also critical to understanding and diagnosing early clinical disease. This group advocates further strategies that enable the prediction and stratification of individual risks for development of future PTOA with an emphasis on an earlier time point, such as at the time of injury. Moreover, they point out that, given that 5–10-year interventional clinical trials are not feasible, more studies need to be done to determine which measurement(s) could act as acceptable surrogate short-term outcome predictors of future PTOA or therapeutic efficacy. This group also suggested that, when possible, samples including serum/plasma, urine and SF should be collected at all available time points, such as preoperatively, at the time of surgery, clinical aspiration, and treatment which, in terms of clinical treatment outcomes, could help identify relationships between early (e.g., 1–2 years) and later outcomes (e.g., 5–10 years). Thus, inclusion of easily measurable biomarkers, combined with imaging technologies, e.g., AI-supported endomicroscopy-assisted optical biopsy, that correlate local tissue architectures [240] with biomarkers and other parameters, such as clinical symptoms, age, sex, BMI and, e.g., the type of injury, could also better stratify joint- or injury-specific progression and improve our understanding of early disease pathology and detection.

The data discussed in this review demonstrated that inflammation and subsequent tissue destruction following trauma to the knee leads to a localized as well as systemic inflammation, much like RA. This persistent long-term low-grade chronic inflammation has already been suggested to be a key mediator in the pathogenesis of OA and a general phenomenon of OA [17,18,242,243] and now we show similarly an important role in knee PTOA. While it is known that serum IL-6 concentrations increase with age and systemic IL-6 appears central to the pathophysiology of physical function decline, as well as many chronic diseases [244,245], “inflammaging”, which is systemic mild inflammation in the absence of an apparent infection associated with the process of biological aging, also contributes to an array of age-related diseases including OA [246,247,248]. In this context, knee PTOA occurs earlier than idiopathic OA but, importantly, causes a similar response. Specifically, IL-6 is already detectable in the plasma within 1 year after knee trauma in young adult to middle-aged patients (age range 43.6 ± 15.1 years) having early clinical PTOA (Kellgren–Lawrence grade 1–2) [39]. While many co-factors could enhance progression towards PTOA, including aging, an early knee trauma predisposes young adolescents and young adults to PTOA [4,236,249,250], suggesting that it could lead to earlier chronic disease and, thus, accelerate the process of “inflammaging”.

In this context and in the consideration of why some patients progress towards disease while other do not, it is important to consider the complex dysregulated interplay between the innate and adaptive immune systems that could occur after knee trauma and how damage-associated molecular patterns (DAMPs) and senescence could tip the balance and incite early uncontrolled inflammation. Under conditions of tissue injury/cellular stress induced by, e.g., mechanical injury, hemorrhage, surgical procedures (e.g., knee injury) and in response to proteolytical release (e.g., MMP-mediated degradation) of ECM, DAMPs are released from the articular cartilage matrix or the native cells themselves. While a full description of DAMPs is outside the scope of this review and could be found elsewhere [17,18,242], we will briefly point out how DAMPs released from cartilage matrix or from dying cells have been implicated in events that could drive chronic inflammation and could lead to systemic knee PTOA. DAMPs from damaged cartilage ECM, such as the 32-mer aggrecan fragment [251,252], small leucine-rich proteoglycans (SLRPs) decorin that functions to increase the retention of aggrecan in the ECM [253], biglycans [254], low MW hyaluronic acid, fibronectin [255,256], and prelamin (the precursor of lamin A) [257] as well as intracellular DAMPs (also known as alarmins) that are extracellularly released either passively or by exocytosis including the high mobility group box 1 (HMGB1) [258,259] and uric acid, the final metabolic product of purine catabolism released from dying cells [67], have been implicated in articular cartilage destruction, inflammation and PTOA disease progression. These DAMPs are capable of activating a broad range of pattern-recognition receptors (PRR), e.g., Toll-like receptors (TLRs) or the cytoplasmic nucleotide-binding oligomerization domain-like receptor pyrin domain-containing-3 (NLRP3) inflammasome, present on or in various cells including chondrocytes, synoviocytes, fibroblasts, monocytes and macrophages that, in turn, trigger a range of inflammatory responses and chemotactic activity through various DAMP-PRR receptor signaling mechanisms. Therefore, the recognition of DAMPs not only by local cells within the joint, but also by recruited effector cells of the immune system, could lead to a hyper-inflammatory response.

Since DAMP-PRR signaling is also needed in tissue repair, it is difficult to decipher how the complexity of multiple DAMP-PRR signaling mechanisms contribute towards an imbalance in inflammatory signaling responses and, more specifically, how this change tissue repair to maladaptive in the knee joint but recent studies suggest that senescence could play a role. Articular cartilage tissue and chondrocytes of the knee joint express significantly more DAMPs (e.g., HMGB-1 and alarmins S100A8 and S100A9) than the hip joint. DAMPs are present in normal healthy chondrocytes, but increased in OA [260]. DAMPs also induce cellular senescence and are produced by senescent cells (SnCs) and, like DAMPs, cellular senescence has been suggested to contribute to low grade chronic systemic inflammation in OA [261,262,263] and in PTOA [189,264,265,266]. Senescent cells (SnCs) increase in response to aging but also increase upon injury, stress and inflammation and, thus, are relevant to trauma-related pathologies. The process of cellular senescence causes irreversible cell-cycle arrest and distinct morphological and phenotypic alterations resulting in adoption of a pro-inflammatory bio–active secretome phenotype, referred to as the “senescence-associated secretory phenotype” (SASP). SnCs release a wide-range of proteins, including DAMPs, as well as pro-inflammatory cytokines, MMPs, and chemokines, including those factors shown in Figure 1 that were significantly increased following trauma to the knee joint, such as IL-6, IL-1, TNF-α and MMP-3. Moreover, inflammatory cytokines present after knee trauma, such as IL-1β, TNF-α and IL-17, drive chondrocytes [267] and fibroblasts [189] towards a senescent phenotype.

As with DAMP-PRR signaling, SnCs are needed for normal healing in acute inflammation and their presence limits fibrosis during tissue repair [268,269]. On the other hand, SnCs are also capable of promoting OA disease progression when transplanted into normal knee healthy joints of mature adult mice equivalent in age to 20–30-year-old humans [261] and in young animals in PTOA knee models [189,264]. Thus, ACLT injury induced senescence in both young and aged animals. The presence of SnCs increased with tissue severity, but also differed in the localization between young and old animals with SnCs localized in the superficial layer of articular cartilage in young animals vs. throughout the cartilage matrix in aged animals. Moreover, treatment with senolytic drugs in both young as well as aged mice protected against inflammation and helped eliminate SnCs and their resulting SASP which, in turn, enabled tissue recovery and the development of ACLT-induced PTOA [189,264]. The study of Faust et al. has shed some light on how this process causes a local Th17 immune response in the articular compartment of the joint and systemic chronic inflammation via the draining inguinal lymph nodes [189]. Their data suggest that high IL-6 or TGF-β after injury skews naïve CD4+ T cells towards Th17 cells and that these Th17 cells contribute to chronic disease. Hence, they suggest that these Th17 cells induce fibroblasts to become senescent and at the same time this in turn could further promote a Th17 phenotype in innate lymph cells resulting in a positive feedback loop and chronic inflammation. Moreover, many DAMP markers have been recognized as SASP markers. Others have shown that extracellular vesicles released from OA senescent chondrocytes are capable of transmitting the senescent phenotype to nearby cells and thereby inhibiting ECM deposition by healthy chondrocytes [270] and the number of SnCs positively correlated with OA severity [271]. It has also been shown that physiological loading increases the diffusion of inflammatory cytokines into injured articular cartilage [272]. These mechanisms could induce senescence in nearby cells and, along with the circulating Th17 cells [189], could reinforce the long-term production of the inflammatory, such as the prominent “inflammaging” IL-6 [244,245], and tissue-degenerating molecules that are increased in those with knee trauma, as shown in our comprised Figure 1. Thus, increased DAMPs that are released from damaged and apoptotic or necrotic cells could induce immunosenescent programming in damaged articular cartilage tissue. If the clearance of SnCs is diminished in certain individuals (possibly the 23–50% of those who progress to PTOA [2,3,4,5,6,7,8]), an amplifying loop of senescent-induced inflammation may accelerate the process of “inflammaging”.

These data strongly emphasize the need to improve our understanding of the early mechanisms that result in an immune response shift and provocation towards the sustained and chronic inflammatory disease that occur following knee trauma, with particular emphasis on the low-grade pro-inflammatory state in healthy and hence young to middle-aged adults. Moreover, as the molecular mechanisms and signaling pathways of these processes and how, e.g., senolytic agents, regulate this is still not clear; further context-specific investigations are needed. Importantly, other co-morbidities, such as subsequent knee injuries [249], abnormal mechanical loading [273,274] following the primary injury, gender [275] (e.g., hormonal) differences, co-morbidities, such as high BMI [249], or the presence of other diseases, such as diabetes [276,277,278] and even genetic risk loci [279,280], that could predispose one to early chronic disease, also need to be considered in pre-clinical injury/inflammatory models to determine how they enhance the early development of chronic inflammation and reduce tissue repair capacity in the setting of PTOA progression. In this context, studies would benefit from focusing on acute vs. chronic models of inflammation, but also healthy vs. different diseased states of cells and tissues, which may provide more insight on the effect of the “starting point” on disease outcome. Patient stratification, and co-morbidity studies as well as biobank data could help in answering these questions.

In summary, chronic inflammation associated with knee trauma, which together lead to PTOA, cannot yet be clinically prevented. Although knowledge and awareness have increased in the past few years, there are many future challenges, yet also multiple promising avenues. Using the Bradford Hill Framework, we show that TNF-α and IL-6 cytokines are causal factors, while IL-1β and IL-17 are credible factors in inducing progression towards knee PTOA. Recently, three out of four of these cytokines, namely IL-1β, TNF-α, and IL-6, have been linked to inducing a dedifferentiated phenotype by converging on signaling pathways that regulate cytoskeletal actin dynamics [281], suggesting a potential link between post-injurious inflammation and a disease-driving phenotype. This may potentially open up a novel avenue of disease intervention. Since PTOA occurs in younger patients compared to other forms of OA, it is evident that early treatment strategies, as opposed to end-stage early knee replacement, are needed to control inflammation before clinical PTOA onset. The future lies not only in regulating the inflammatory aspects of this challenging disease, but more so in recognizing a PTOA-specific immunopathology at a much earlier stage, as this would ultimately allow for the development of clinical preventative interventions.

## Figures and Tables

**Table 1:** Quality assessment results of the clinical observational cohort and cross-sectional knee injury studies included in this review. Quality was assessed using the (NIH) Study Quality Assessment Tool and guidelines. Abbreviations: Question (Q), yes (Y), no (N), not applicable (NA) or not reported (NR). Yes answers are given a score of 1 and no answers a score of 0. The study quality is defined as 0–5 (poor); 6–9 (fair): 10–14 (good).

Study	Q1	Q 2	Q 3	Q 4	Q 5	Q 6	Q 7	Q 8	Q 9	Q 10	Q 11	Q 12	Q 13	Q 14	Score	Quality
Haller [21]	1	1	0	1	0	1	1	1	1	NA	1	1	1	0	10	good
Haller [22]	1	1	0	1	0	1	1	1	1	NA	1	1	1	0	10	good
Swärd [23]	1	1	1	0	0	1	1	1	1	NA	1	NR	1	1	10	good
Bigoni [24]	1	1	1	1	0	1	1	1	1	NA	1	NR	1	0	10	good
Irie [25]	1	1	1	1	0	1	1	1	1	NA	0	NR	0	0	8	fair
Elsaid [26]	1	1	1	1	1	1	1	1	1	NA	0	NR	0	1	10	good
Catterall [27]	1	1	1	0	0	1	1	0	1	NA	0	NR	1	0	7	fair
Watt [28]	1	1	1	1	1	1	1	1	1	NA	1	NR	1	1	12	good
Lattermann [29]	1	1	1	0	0	1	1	0	1	NA	1	NR	U	0	7	fair
Dahlberg [30]	1	1	1	1	0	1	1	1	1	NA	1	NR	1	0	10	good
Higuchi [31]	1	1	1	0	0	1	1	1	1	NA	1	NR	1	0	9	fair
Struglics [32]	1	1	1	0	U	1	1	1	1	NA	0	U	1	1	9	fair
Sarafan [33]	1	0	1	U	0	1	1	0	1	NA	1	NR	1	0	7	fair
Lohmander [34]	1	1	0	0	0	1	1	1	1	NA	1	NR	NA	1	8	fair
Lohmander [35]	1	1	0	0	0	1	1	1	1	NA	1	NR	NA	1	8	fair
Struglics [36]	1	1	1	0	0	1	1	1	1	NA	1	NR	1	1	10	good
Struglics [37]	1	1	0	1	1	1	1	1	1	NA	1	NR	0	1	10	good
Larsson [38]	1	1	NA	1	0	1	1	1	1	NA	1	1	1	0	10	good
Panina [39]	1	1	1	1	0	1	1	1	1	NA	0	NR	1	1	10	fair

**Table 2:** Details of the clinical observational cohort and cross-sectional knee injury studies included in this review. The phase after injury refers to acute (A), subacute (S), chronic (C) inflammation or clinically diagnosed PTOA. For the remaining abbreviations we refer the readers to the list of abbreviations. The age, cohort size and sex are of the injured group only. Data not reported (NR) indicate that the information was not described in the study.

Study	Type of Injuries	Comparison Group	Phase after Injury	Markers Measured	Age Mean Median (Range)	Cohort Size	Sex	Confounding Factor
**Haller** [21]	24 patients had low and 21 high energy acute tibial plateau fracture	Contralateral uninjured knee	A	IFN-γ, IL-1β, IL-1RA, IL-2, IL-4, IL-6, IL-7, IL-8, IL-10, IL-12(p70), IL-13, IL-17A, TNF-α, MCP-1, MIP-1β	42 (20–60)	45	31 males 14 females	
**Haller** [22]	24 patients had low and 21 high energy acute tibial plateau fracture	Contralateral uninjured knee	A	MMP-1, -2, -3, -7, -9, -10, -12, -13	42 (20–60)	45	31 males 14 females	
**Swärd** [23]	100% had hemarthrosis; 57 ACL tear, 3 medial meniscus injuries, 1 posterior cruciate ligament tear, 5 patellar dislocations; 1 suspected tibial fracture; 3 MCL tear, 2 lateral collateral ligament tears, 35 rotational knee traumas; 3 knee contusions; 1 knee hyperextension. 15 of these had associated MCLT	Age- and gender-matched healthy uninjured controls	A, S	sGAG, ARGS aggrecan fragments, OCL, SPARC, OPN, IL-1β, IL-6, IL-8, TNF-α	25 (13–64)	111	83 males 28 females	16 patients had a previous injury (mean 5 years prior to injury) and 7 had previous knee surgery
**Bigoni** [24]	18 patients had ACL tear, 30 ACL tear associated + meniscal injury	Healthy uninjured controls	A, S, C	IL-1, IL-1ra, IL-6, IL-8, IL-10, TNF-α	30 (14–55)	48	48 males	Unequal distribution of the number of patients at the different time points measured; Did not include any female subjects
**Irie** [25]	ACL tear	OA & post- meniscectomy including hydrarthrosis	A, S	TNF-α, IL-1β, IL-6, IL-8, IL-1ra, IL-10	27 (13–55)	34	20 males 14 females	Unequal distribution of the number of patients at the different time points measured
**Elsaid** [26]	ACL tear	Contralateral uninjured knee	S, C	Lubricin, IL-1β, TNF-α, IL-6, Procathepsin B, Neutrophil elastase, sGAG	24 (15–47)	30	19 males 11 females	Unequal distribution of patients at different time points; No sub-stratification of the injured groups based on time after injury
**Catterall** [27]	ACL tear + other knee joint tissue damage including bone contusions, medial collateral ligament tears, meniscal tears and chondral defects	None (samples were measured over time)	A, S	IL-1β, CRP, sGAG, ARGS-aggrecan, FA846, CS846, C2C, CTxII, C1,2C, CTxI, NTX, CPII, Osteocalcin, β-Aspartate, D-Asx, D-Serine, sCD44, COMP, Tenascin C, Lubricin, MMP-3	23 (19–26)	11	6 males 5 females	Half of the patients at 2^nd^ time point were given i.a. IL-1ra but authors reported no statistical differences between IL-1ra vs. placebo arms for all measured biomarkers
**Watt** [28]	74% had hemarthrosis; 61 combined ACL + meniscal tear, 34 severe trauma (1 or more injuries including meniscal tear, cruciate ligament rupture, collateral ligament tear, posterolateral corner injury, traumatic chondral defects, articular or periarticular fracture, or patellofemoral or tibiofemoral dislocation with severe knee trauma defined as combined (>1) ligament rupture, fracture or dislocation); 28 a single ACL or MCL rupture, 27 meniscal tears	Macroscopically intact cartilage from lower limb tumor amputation or transplant donations	A, S, C	Activin A, CRP, IL-1β, IL-6, MCP-1, MMP-3, TIMP-1, TSG-6	25 (16–50)	150	121 males 29 females	Wide time range for baseline visit (0–8 weeks)
**Lattermann** [29]	Isolated ACL injury (no more than a grade 1 MCL injury defined clinically)	None (samples were measured over time)	A, S	IL-1α, IL-1β, IL-1ra, COMP fragments, CTX-II, sGAG, MMP-1, MMP-3, MMP-9, NTX-I, TSG-6	NR (18–32)	41	NR	Controls were not included
**Dahlberg** [30]	31 patients had a combination of cruciate ligament, collateral ligament and meniscus injuries, 15 isolated meniscus injuries, 8 cartilage lesions without ligament injuries; remaining had fibrillations + occasional clefts in the joint cartilage surface in one knee compartment	Contralateral uninjured knee or healthy uninjured controls	A, S, C	Aggrecan fragments, COMP, MMP-3, TIMP-1	28 (18–40)	54	NR	4 patients had undergone ligament reconstruction more than 3 years before
**Higuchi** [31]	Complete ACL rupture	Age-matched healthy uninjured controls	A, S, C	IL-1β, TNF-α, IL-6, MMP-3, TIMP-1	26 (17–42)	32	20 males 12 females	Wide time range for injury group (2–134 weeks) and with no sub-stratification of the injured groups for most of the markers measured; Performed mostly correlations
**Struglics** [32]	Acute ruptured ACL	Healthy uninjured controls	A, S, C	TNF-α, IL-6, IL-8, IL-10, IFN-γ, ARGS, CTX-II, NTX-I	NR (21–31)	121	152 males 31 females	Wide time range for baseline visit (0–6 weeks)
**Sarafan** [33]	Intra-articular fractures	Healthy uninjured controls	C	IL-17, CRP	NR (28–42)	20	9 males 11 females	Confusing inclusion/exclusion criteria; While the authors report that fracture patients endured pain for at least 3 months and samples were taken at the time of knee joint surgery, it was not clear when the samples were collected
**Lohmander** [34]	ACL rupture isolated or combined with another ligament or meniscus tear or an isolated meniscus tear	Healthy uninjured controls	A, S, C	CTX-II, Aggrecan fragments, MMP-1, MMP-3, TIMP-1	37 (14–70)		247 males 82 females	
**Lohmander** [35]	159 patients had ACL rupture +/− injury to meniscus; 129 medial or lateral meniscus tear	Healthy uninjured controls	A, S, C	Aggrecan and COMP fragments, CPII, MMP-1, MMP-3, TIMP-1, BSP	33 (14–70)		221 males 67 females	
**Struglics** [36]	Patients were recently injured (0–12 weeks after injury; 98% had hemarthrosis) or had an old injury (1–37 years after injury): 39 had ACL, 4 PCL, 69 ACL injury + meniscal tear, 37 ACL injury + meniscal tear + other ligament injuries, 47 ACL injury + other ligament injuries, 58 isolated meniscal tear, 7 meniscal tear + non-ACL ligament injuries, 13 patellar dislocation +/− soft tissue injuries, 7 other injuries + medial or lateral collateral ligament tears; 2 + give-way), 10 no signs of soft tissue injury	Healthy uninjured controls, OA, RA or PPA	A, S, C, PTOA	C4d, C3bBbP, sTCC, sGAG, IL-1β, IL-6, IL-8, TNF-α, ARGS neoepitope of aggrecan, Osteocalcin, SPARC, COMP, C2C, Osteopontin	Recently injury 26 (13–64) Old injury 32 (18–65)	219 75	164 males 55 females 50 males 25 females	Only 8% of the old injury group were diagnosed with PTOA; Sub-stratification of the group was only divided into two groups: 1–3 years (median of 2.0 years) and 3.01–36.9 years (median of 5.1 years) with the latter group having a wide range of time after injury
**Struglics** [37]	ACL injury	Healthy uninjured controls	A, S, C	COMP	26 (21–31)	121	90 males 31 females	
**Larsson** [38]	ACL rupture +/− meniscus tear	Healthy uninjured controls	A, S, C, PTOA	Aggrecan, ARGS aggrecan fragments, sGAG	Acute knee injury 27 (16–59) Chronic knee injury 40 (16–70)	192	56 males 13 females 95 males 28 females	Chronic knee injury group had a wide range of time from 3 months–36 years (mean of 1 year) after injury and 97% had PTOA
**Panina** [39]	All patients suffered a knee injury ≤ 1 year following a meniscus injury and were diagnosed with early- (Kellgren–Lawrence grade 1–2) or late-stage (grade 3–4) PTOA	Healthy uninjured controls	PTOA	IL-1β, IL-6, IL-18, TNF-α, Leptin, NO	NR (26–65)	134	60 males 74 females	77% of patients were in the early-stage PTOA group; Control data was only included for plasma samples; not for SF

**Table 3:** Concentrations of pro-inflammatory cytokines measured in the synovial fluid after knee trauma or in clinically diagnosed cases of knee OA vs. those used in in vitro chondrocyte and ex vivo cartilage explant models.

Cytokine	Concentration in Synovial Fluid	Phase Present after Knee Trauma	Concentration Used in Laboratory Models	Fold Change
**IL-1β**	0–25 pg/mL	Acute, Subacute, PTOA	25–1 × 10^5^ pg/mL	50–400×
**TNF-α**	7–20 pg/mL	Acute, Subacute, Chronic	100–1 × 10^6^ pg/mL	77–5000×
**IL-6**	1–66,099 pg/mL	Acute, Subacute, Chronic, PTOA	100–2 × 10^6^ pg/mL	3–5×
**IL-17**	2–8 pg/mL	Chronic, PTOA	100–1 × 10^6^ pg/mL	12–52×

**Table 4:** Use of the Bradford Hill criteria as evidence of causation between the presence of IL-1β, TNF-α, IL-6 or IL-17 after knee trauma and a PTOA disease progressive effect. We used this as a framework to evaluate the relationship between the presence of inflammatory markers (IL-1β, TNF-α, IL-6, or IL-17) at different stages of inflammation after knee trauma to determine whether these markers led to a convincing, credible or probable causal PTOA disease progressive effect or whether the evidence was suggestive, limited, or inconclusive.

		IL-1β	TNF-α	IL-6	IL-17
**STRENGTH OF ASSOCIATION**	Association between risk factor and outcome	**Convincing** [21,23,24,25,26,27,29,31,39,208]	**Convincing** [23,24,25,26,28,32,36,37]	**Convincing** [21,23,24,25,26,28,31,32,36,37,39,208,209]	**Convincing** [21,33,55,56,57,58,59,60]
**CONSISTENCY OF FINDINGS**	The same findings are observed among different locations, populations or in different study designs including different types of injuries	**Convincing** While some studies showed that IL-1β was significantly increased vs. [21,23,24,25,26,27,29,31,39], some studies showed that it was below the detection for some patients [24,26,28,29,31]	**Convincing** [23,24,25,26,28,32,36,37]	**Convincing** [21,23,24,25,26,28,31,32,36,37,39]	**Convincing** [21,33]
**SPECIFICITY OF ASSOCIATION**	The factor influences the outcome	**Credible** Clinically present from 24 h to 1.5 months in A, S phases [21,23,24,25,26,27,29,31] and in early PTOA [39] indicating that it influences disease progression Conflicting data: Some studies showed that IL-1β was not present [28] or only present in some patients [24,26,29,31] Application of IL-1β in vivo, ex vivo & in vitro mirrors clinical symptoms (Figure 2A–C)	**Convincing** Clinically present from 24 h to 5 years in A, S, C phases [23,24,25,26,28,32,36,37] Application of TNF-α in vivo, ex vivo and in vitro mirrors clinical symptoms (Figure 2A–C)	**Convincing** Clinically present from 24 h to 1 year in A, S, C phases and in early & late PTOA [21,23,24,25,26,28,31,32,36,37,39] IL-6 KO mice develop more advanced knee OA [210] and application of IL-6 ex vivo and in vitro mirrors clinical symptoms (Figure 2A–C)	**Credible** There are only 2 clinical studies showing that IL-17 is present from 24h to 9.5 days in the A phase [21] and C phase (3 or more months) after injury [33] Application of IL-17 in vivo, ex vivo and in vitro mirrors clinical symptoms (Figure 2A–C)
**TEMPORAL SEQUENCE OF ASSOCIATION**	The factor precedes the outcome	**Convincing** A, S, Early PTOA [21,23,24,25,26,27,29,31,39]	**Convincing** A, S, C Phases [23,24,25,26,28,32,36]	**Convincing** A, S, C, Early & Late PTOA [21,23,24,25,26,28,31,32,36,39]	**Convincing** A, C Phases [21,33]
**BIOLOGICAL GRADIENT**	Dose-response relationship, where longer or higher exposure leads to an increased risk of disease	**Convincing** Early PTOA was associated with a highIL-1β plasma concentration [39]	**Convincing** Correlationbetween TNF-α concentrations and ARGS fragments in the SF up to 5 years after knee trauma [32] suggesting that long exposure to TNF-α increases the risk of disease	**Convincing** Early and late PTOA were associated with high IL-6 concentrations PTOA [39]	**Convincing** Levels of IL-17 in the SF correlate with severity of knee OA [58] Presence of IL-17 in synovial fluid identifies a subset of patients with end-stage knee OA [60]
**BIOLOGICAL PLAUSIBILITY**	Presence of a potential biological mechanism	**Convincing** [12,211]	**Convincing** [12,212]	**Convincing** [191,213]	**Convincing** [187,189]
**COHERENCE**	The current studies agree and do not conflict with previously reported evidence and the biology of disease	**Credible** Clarification is needed on why some patients have high concentrations of IL-1β present, while others do not	**Convincing**	**Convincing**	**Credible** More clinical studies need to measure IL-17 in the SF and serum at the various phases after knee trauma and in clinical PTOA
**EXPERIMENTAL EVIDENCE**	Evidence drawn from experimental models agree with clinical data	**Convincing**	**Convincing**	**Convincing**	**Convincing**
**ANALOGY**	Analogous examples that lead to the same outcome	**Convincing** RA, periodontitis [214,215]	**Convincing** RA, psoriatic arthritis ankylosing spondylitis, Crohn’s disease [216,217]	**Convincing** RA, psoriatic arthritis, periodontitis [216,218,219]	**Convincing** RA, psoriatic arthritis, ankylosing spondylitis, periodontitis [184,216,220,221]
**CAUSAL EFFECT**		**7/9** **EVIDENCE MET**	**9/9** **EVIDENCE MET**	**9/9** **EVIDENCE MET**	**7/9** **EVIDENCE MET**

## Abbreviations

ACAN	Aggrecan
ACI	Autologous chondrocyte implantation
ACL	Anterior cruciate ligament
ACLT	Anterior cruciate ligament transection
ADAMTS	A disintegrin and metalloproteinase with thrombospondin motifs
AI	Artificial intelligence
ARGS	Alanine–Arginine–Glycine–Serine
BMI	Body mass index
C1,2C	Type I/II collagen degradation
C2C	Type II collagen cleavage product
C2M	MMP-mediated type II collagen
CCL2	CC-chemokine ligand 2, also known as MCP-1
CH	Chondrocyte
CHI3L1	Chitinase 3-like 1
CLU	Clusterin
COLI	Collagen I
COLII	Collagen II
COMP	Cartilage oligomeric matrix protein
CPII	Procollagen II c-propeptide
CRP	C-reactive protein
CS	Chondroitin sulphate
CTX-I	C-terminal crosslinked telopeptide type I collagen
CTX-II	C-terminal crosslinked telopeptide type II collagen
CXCL	Chemokine ligand
DAMPs	Damage-associated molecular patterns
ECM	Extracellular matrix
FB	Factor B
FRI	Fluorescence reflectance imaging
gp 130	Glycol–protein 130
HMGB1	High mo­bility group box 1
IFN-γ	Interferon gamma
IL	Interleukin
IL-1ra	IL-1 receptor antagonist
IL-6R	IL-6 receptor
iNOS	Inducible NO synthase
KOOS4	Knee Injury and Osteoarthritis Outcome Score 4
LIF	Leukemia inhibitor factor
LPS	Lipopolysaccharide
MASP	MBL-associated serine proteases
MBL	Mannose binding lectin
MCL	Medial collateral ligament
MSC	Mesenchymal stem cells
MCP-1	Monocyte chemoattractant protein-1, also known as CCL2
MIP-1β	Macrophage inflammatory protein-1 beta, also known as CCL4
MIP-3α	Macrophage inflammatory protein-3 alpha, also known as CCL20
MMPs	Matrix metalloproteinases
MMX	Partial medial meniscectomy
MNC	Mononuclear cells
MRI	Magnetic resonance imaging
NLRP3	Nucleotide-binding oligomerization domain-like receptor pyrin domain-containing-3
NO	Nitric oxide
NTX-1	N-terminal telopeptides of type I collagen
OA	Osteoarthritis
OCL	Osteocalcin
OPN	Osteopontin
PG	Proteoglycan
PLGA	Poly (lactic-co-glycolic acid)
PPA	Pyrophosphate arthritis, also known as pseudogout
PRP	Platelet rich plasma
PRR	Pattern-recognition receptors
PTOA	Post-traumatic osteoarthritis
RA	Rheumatoid arthritis
SASP	Senescence-associated secretory phenotype
SCSO	Superficial zone chondrocytes
SF	Synovial fluid
sGAG	Sulfated glycosaminoglycan
SPARC	Secreted protein acidic and rich in cysteine, also known as osteonectin
sTCC	Soluble terminal complement complex
SnCs	Senescent cells
TIMP	Tissue inhibitor of metalloproteinases
TLRs	Toll-like receptors
TNF-α	Tumor necrosis factor alpha
TSG-6	Product of tumor necrosis factor alpha-stimulated gene 6
Th17	T helper 17
VEGF	Vascular endothelial growth factor
